# Downregulation of RdDM during strawberry fruit ripening

**DOI:** 10.1186/s13059-018-1587-x

**Published:** 2018-12-04

**Authors:** Jingfei Cheng, Qingfeng Niu, Bo Zhang, Kunsong Chen, Ruihua Yang, Jian-Kang Zhu, Yijing Zhang, Zhaobo Lang

**Affiliations:** 1 National Key Laboratory of Plant Molecular Genetics, CAS Center of Excellence in Molecular Plant Sciences, Institute of Plant Physiology and Ecology, Shanghai Institutes for Biological Sciences, Chinese Academy of Sciences, Shanghai, 200032 China; 2 University of the Chinese Academy of Sciences, Beijing, 100049 China; 3 Shanghai Center for Plant Stress Biology, National Key Laboratory of Plant Molecular Genetics, Center of Excellence in Molecular Plant Sciences, Shanghai Institutes for Biological Sciences, Chinese Academy of Sciences, Shanghai, 200032 China; 4 Laboratory of Fruit Quality Biology/Zhejiang Provincial Key Laboratory of Horticultural Plant Integrative Biology, Zhejiang University, Zijingang Campus, Hangzhou, 310058 China; 5 Horticultural Department, Heilongjiang Academy of Agricultural Sciences, Harbin, China; 6 Department of Horticulture and Landscape Architecture, Purdue University, West Lafayette, IN 47907 USA

**Keywords:** DNA methylation, Fruit ripening, Strawberry, RdDM, siRNA

## Abstract

**Background:**

Recently, DNA methylation was proposed to regulate fleshy fruit ripening. Fleshy fruits can be distinguished by their ripening process as climacteric fruits, such as tomatoes, or non-climacteric fruits, such as strawberries. Tomatoes undergo a global decrease in DNA methylation during ripening, due to increased expression of a DNA demethylase gene. The dynamics and biological relevance of DNA methylation during the ripening of non-climacteric fruits are unknown.

**Results:**

Here, we generate single-base resolution maps of the DNA methylome in immature and ripe strawberry. We observe an overall loss of DNA methylation during strawberry fruit ripening. Thus, ripening-induced DNA hypomethylation occurs not only in climacteric fruit, but also in non-climacteric fruit. Application of a DNA methylation inhibitor causes an early ripening phenotype, suggesting that DNA hypomethylation is important for strawberry fruit ripening. The mechanisms underlying DNA hypomethylation during the ripening of tomato and strawberry are distinct. Unlike in tomatoes, DNA demethylase genes are not upregulated during the ripening of strawberries. Instead, genes involved in RNA-directed DNA methylation are downregulated during strawberry ripening. Further, ripening-induced DNA hypomethylation is associated with decreased siRNA levels, consistent with reduced RdDM activity. Therefore, we propose that a downregulation of RdDM contributes to DNA hypomethylation during strawberry ripening.

**Conclusions:**

Our findings provide new insight into the DNA methylation dynamics during the ripening of non-climacteric fruit and suggest a novel function of RdDM in regulating an important process in plant development.

**Electronic supplementary material:**

The online version of this article (10.1186/s13059-018-1587-x) contains supplementary material, which is available to authorized users.

## Background

Methyl-cytosine (mC) is a conserved epigenetic mark in many eukaryotic organisms and is involved in a wide range of biological processes, such as gene regulation, immunity, imprinting, and genome stability [1, 2]. Methylation of cytosine occurs in three sequence contexts: symmetric CG, CHG, and asymmetric CHH (H=A, C, or T). In *Arabidopsis*, DNA methylation in CG and CHG can be maintained by METHYLTRANSFERASE 1 (MET1) and CHROMOMETHYLASE 3 (CMT3) respectively, while CHH methylation is mainly maintained by CMT2 [2, 3]. De novo DNA methylation in all three contexts can be established by DOMAINS REARRANGED METHYLASEs (DRMs) through the RNA-directed DNA methylation (RdDM) pathway. In RdDM, there are two main steps: siRNA biogenesis and siRNA-guided DNA methylation. Pol IV (RNA polymerase IV) and DCLs (DICER-LIKES) are involved in the first step, while Pol V, AGO4/6 (ARGONAUTE 4/6), and DRMs are involved in the second step [4, 5]. DNA methylation status can be dynamically regulated by both DNA methyltransferases and demethylases. The DNA demethylases, REPRESSOR OF SILENCING 1 (ROS1) family proteins, have DNA glycosylase/lyase activities and thus can actively initiate DNA demethylation process by removal of the mC base and cleavage of the DNA backbone at the abasic site, leaving a single-nucleotide gap that is later filled with a non-methylated cytosine [1, 6].

Fruit, the angiosperm-specific developmental structure that facilitates seed dispersal, constitutes an important source of human daily diet. The major fruit types include dry fruits, such as nuts, and fleshy fruits, such as peaches. The development of fleshy fruit has three main stages: cell division, cell expansion, and ripening. Fleshy fruits can be further classified as climacteric and non-climacteric, based on their ripening process. The ripening of climacteric fruits, such as tomatoes, is accompanied with increasing ethylene production and respiration bursts, while the ripening of non-climacteric fruits is not [7]. Most studies about ripening mechanisms have been carried out in tomato, a classical climacteric fruit. For the study of non-climacteric fruits, strawberry has been used as a model plant. The ripening of climacteric fruits mainly depends on the phytohormone ethylene. In contrast, the phytohormone abscisic acid (ABA) plays a more prominent role in the ripening of non-climacteric fruits, such as strawberries. Indeed, suppression of 9-cis-epoxycarotenoid dioxygenase (*NCED*), a vital enzyme in ABA biosynthesis, can cause a severe delay in strawberry ripening [8].

Emerging evidence suggests that DNA methylation also plays an important role in fleshy fruit ripening. In tomato, a naturally occurring epimutation with hypermethylation in the *COLORLESS NON-RIPENING* (*CNR*) promoter results in abnormal fruit ripening [9]. In apple, DNA methylation of the *MdMYB10* promoter regulates gene expression and fruit pigmentation during ripening [10, 11]. In addition, tomatoes undergo global DNA hypomethylation during ripening due to the increased expression of a DNA demethylase gene, and mutations in the DNA demethylase can inhibit ripening [12–14]. Genome-wide DNA methylation dynamics have not been investigated in non-climacteric fruits.

Technological advances have enabled the sequencing and assembly of the genomes for many plant species, including that of the diploid woodland strawberry (*Fragaria vesca*) [15]. In addition, whole-genome bisulfite sequencing has facilitated the analysis of genome-wide DNA methylation profiles [16], and the DNA methylomes of 34 flowering plants, including leaf tissue from the diploid woodland strawberry, were recently reported [17]. Given that strawberry is an economically important crop, the DNA methylome of strawberry fruit, especially the DNA methylation dynamics during ripening, is of interest. To investigate the epigenetic regulation of non-climacteric fruit ripening, we characterized the DNA methylomes, genome-wide siRNA profiles, and transcriptomes of octoploid cultivated strawberry fruit (*Fragaria x ananassa*) at different stages of ripening. We observed global DNA demethylation in ripe fruit compared to immature fruit, similar to that observed during tomato ripening. The application of a DNA methylation inhibitor caused hypomethylation and early ripening, suggesting that the ripening-induced decrease in DNA methylation is important for the normal fruit ripening process. We discovered that DNA hypomethylation during strawberry ripening is associated with decreased expression of genes encoding components in the RNA-directed DNA methylation (RdDM) pathway and with decreased siRNA levels. The *Tobacco rattle virus* (TRV)-induced gene silencing of an important RdDM component, *FvAGO4*, leads to an early ripening phenotype in strawberry fruit. It is noteworthy that hundreds of ripening-regulated genes display DNA hypomethylation in their promoters, which helps to explain the epigenetic regulation of strawberry fruit ripening. In summary, our study for the first time reveals the dynamics of DNA methylation in non-climacteric fruit ripening and suggests a distinct mechanism for the loss of DNA methylation during fruit ripening.

## Results

### The DNA methylome of strawberry fruit

**Fig. 1:**
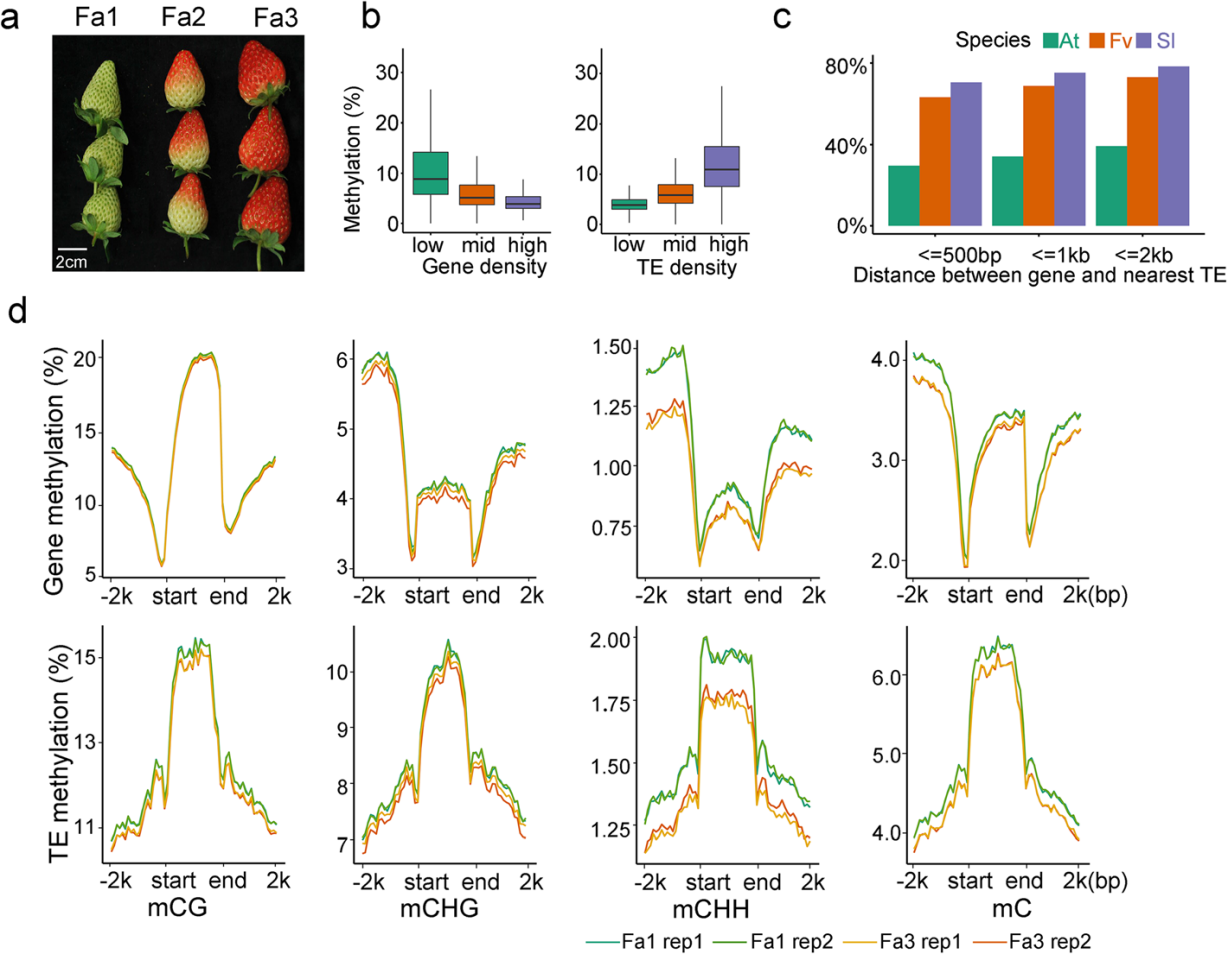
Characterization of strawberry methylomes. **a** Picture of strawberry fruits at different stages. Immature (Fa1), half-red (Fa2), and fully red (Fa3) fruits were used. **b** Correlation between DNA methylation level and gene (left panel) or TE densities (right panel). **c** Percentages of genes that have TEs within 500 bp, 1 kb and 2 kb in *Arabidopsis* (At), strawberry (Fv), and tomato (Sl) genomes, respectively. **d** DNA methylation profiles of mCG, mCHG, mCHH and mC surrounding genes (upper panel) and TEs (lower panel) in immature (Fa1) and fully red (Fa3) fruits. Two biological replicates were shown. Transcription start site (TSS) and transcription end site (TES) are indicated

To characterize strawberry methylomes, we performed whole-genome bisulfite sequencing and generated single-base resolution maps of DNA methylation for both leaf and fruit tissues of *F. ananassa*. Fruits from immature to ripe stages (Fa1-Fa3) were sequenced with two biological replicates (Fig. 1a). Due to the high level of collinearity between cultivated octoploid strawberry (*F. ananassa*) and wild diploid strawberry (*F. vesca*), and the lack of a well-assembled genome for *F. ananassa*, we used the genome of *F. vesca* as reference [15, 18] in our analyses. The genome of *F. vesca* is about 240 MB (2*n* = 14). For each sequencing library, at least 100 M paired-end reads (read length = 150 bp) were produced, covering > 80% of the genome. For each bisulfite-treated library, ~ 10% of the total reads were mapped to the unmethylated chloroplast genome, and the conversion rates were > 99.6% for all libraries (Additional file 1: Table S1). Each methylome was sequenced with an average > 10-fold coverage per DNA strand. The sequencing coverage and depth are comparable to those of published methylomes of *Arabidopsis* and tomato [14, 19].

Immature strawberry fruit displayed an average DNA methylation of about 7.5%, which is lower than that of tomato fruit (22%) [14], and the average methylation levels of mCG, mCHG, and mCHH were 40, 11, and 2%, respectively. In plants, transposable elements (TEs) and repeats are usually highly methylated. To investigate the genomic distribution of DNA methylation in strawberry, we annotated TEs and repeats in the *F. vesca* genome de novo using RepeatScout [20]. TE and repeat contents in the strawberry genome were higher than those of *Arabidopsis*, but lower than those of rice and tomato (Additional file 2: Figure S1a), consistent with proposals that larger genomes tend to have higher repeat contents [21]. Further, TEs and repeats, as well as DNA methylation, in the strawberry genome were not obviously concentrated in pericentromeric regions (Additional file 2: Figure S1b), unlike in *Arabidopsis* and tomato genomes, possibly because the strawberry genome is not as well-assembled as the *Arabidopsis* and tomato genomes, particularly in the pericentromeric regions. However, similar to *Arabidopsis* and tomato, DNA methylation was high in TE- and repeat-rich genomic regions, and low in gene-rich regions in strawberry fruit (Fig. 1b). TEs and repeats were methylated in all three contexts, whereas gene bodies were enriched with mCG but depleted of non-CG methylation (Additional file 2: Figure S1c).

Leaves displayed an average mC level of about 8%, slightly higher than that of immature fruits (7.5%). Leaves and fruit exhibited similar DNA methylation patterns around genes and TEs, but in general, leaves have a higher DNA methylation level relative to fruits (Additional file 2: Figure S1c). According to a previous study, genes in medium-sized genomes, such as tomato, have shorter distances to the nearest TEs compared to those in small genomes such as *Arabidopsis* and thus are more likely regulated by methylation changes of nearby TEs [14]. The distances between genes and the closest TEs in the strawberry genome are shorter than that in *Arabidopsis*, but longer than that in tomato (Fig. 1c), suggesting that strawberry genes might be more vulnerable to nearby methylation changes than *Arabidopsis* genes.

### Loss of DNA methylation during strawberry ripening

**Fig. 2:**
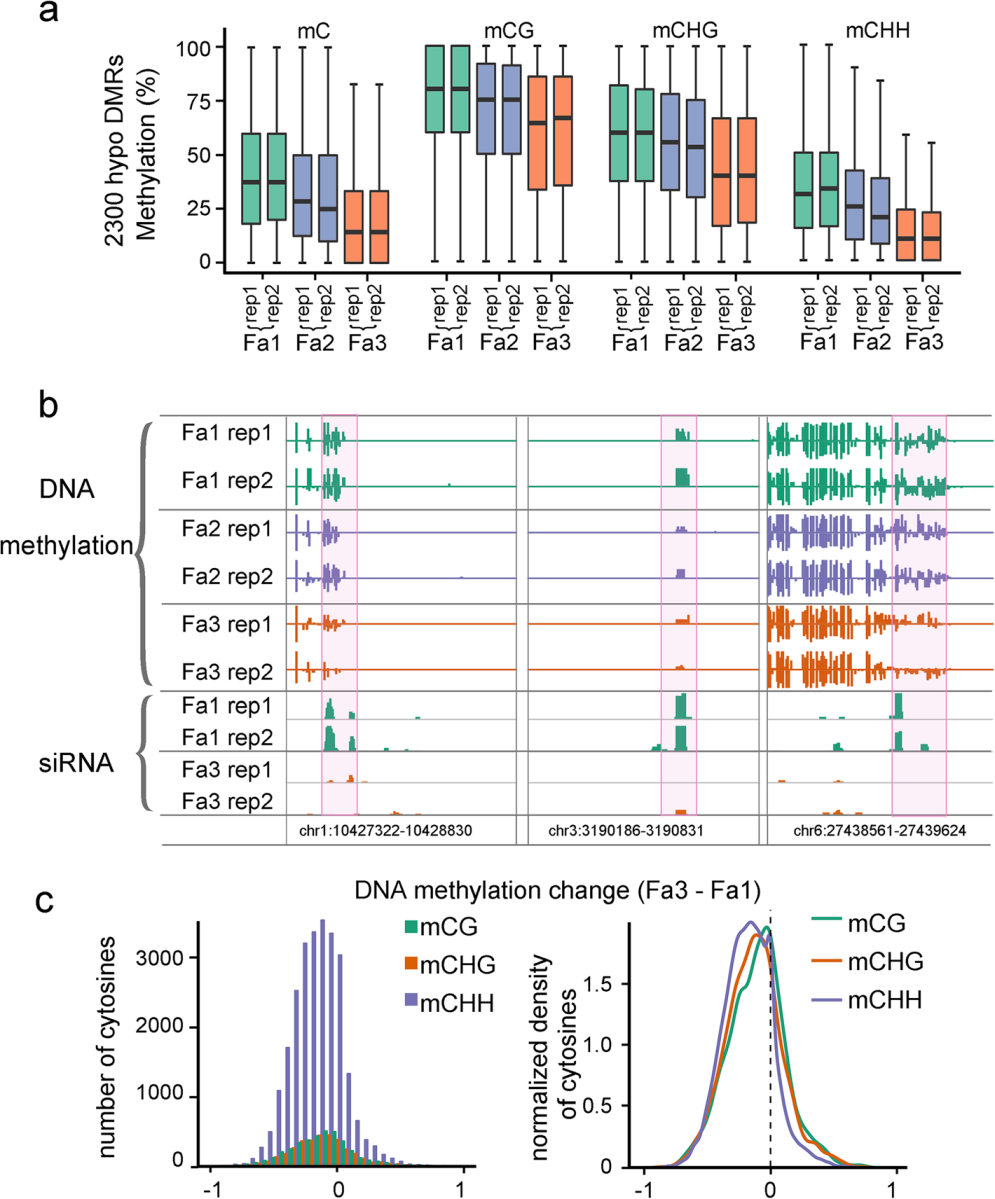
DNA methylation dynamics during strawberry ripening. **a** Boxplots showing DNA methylation levels of 2300 hypo-differentially methylated regions (DMRs) in Fa3 relative to Fa1 in all stages. Methylation levels in mC, mCG, mCHG, and mCHH contexts of two biological replicates are shown. **b** Integrated Genome Browser (IGB) display of DNA methylation levels and 24-nt siRNA levels of representative hypo-DMRs. DNA methylation levels of cytosines and siRNA levels are indicated by the heights of the vertical bars on each track. Genome coordinates are indicated at the bottom. Two biological replicates are shown. **c** Distribution of ripening-induced methylation change of cytosines in different contexts. Cytosines within 2766 DMRs in Fa3 relative to Fa1 were used. DNA methylation change (Fa3-Fa1) for each cytosine was plotted according to the number of cytosines

DNA methylation decreases dramatically during the ripening of tomato, a typical climacteric fruit. To investigate DNA methylation dynamics during ripening of a non-climacteric fruit, we compared the DNA methylomes of strawberry fruit at three different stages (Fa1-Fa3), with two biological replicates for each stage (Fig. 1a). Principal component analysis (PCA) showed consistency between two biological replicates at each stage (Additional file 2: Figure S2a). We found that, in general, ripe fruits have a lower DNA methylation level than immature fruits around genes and TEs (Fig. 1d), suggesting a decrease in DNA methylation during ripening. This decrease occurred in both biological replicates (Fig. 1d). To identify ripening-induced differentially methylated regions (DMRs), we compared the methylomes of Fa1 and Fa3 fruits. Because the two biological replicates were highly consistent, we combined the data from the two biological replicates to increase the statistical power of DMR calling. We found a total of 2766 DMRs in Fa3 compared to Fa1, among which 466 were hypermethylated (hyper-DMRs) and 2300 were hypomethylated (hypo-DMRs) (Additional file 3: Table S2). The DNA methylation changes showed a high correlation between biological replicates (cor = 0.58, Additional file 2: Figure S2b). The larger number of hypo-DMRs vs. hyper-DMRs also suggests a decrease in DNA methylation during strawberry ripening. To investigate whether the change of methylation preferentially occurred in any sequence context, we examined DNA methylation levels of hypo-DMRs and hyper-DMRs in the two biological replicates in CG, CHG, and CHH, respectively. As shown in Fig. 2a and Additional file 2: Figure S2c, the two biological replicates showed the same trend of change that DNA methylation was gradually changed in all three contexts from Fa1 to Fa3 (Fig. 2a, Additional file 2: Figure S2c). The methylation levels of several representative hypo- and hyper-DMRs are shown in Fig. 2b, Additional file 2: Figure S2d-e. We calculated the DNA methylation levels for each cytosine in all three contexts within all DMRs, which showed a left-skewed pattern, suggesting that most cytosines were hypomethylated in Fa3 compared to Fa1 (Fig. 2c). Together, these analyses suggest that strawberries undergo an overall loss of DNA methylation during ripening, similar to tomatoes [12].

**Fig. 3:**
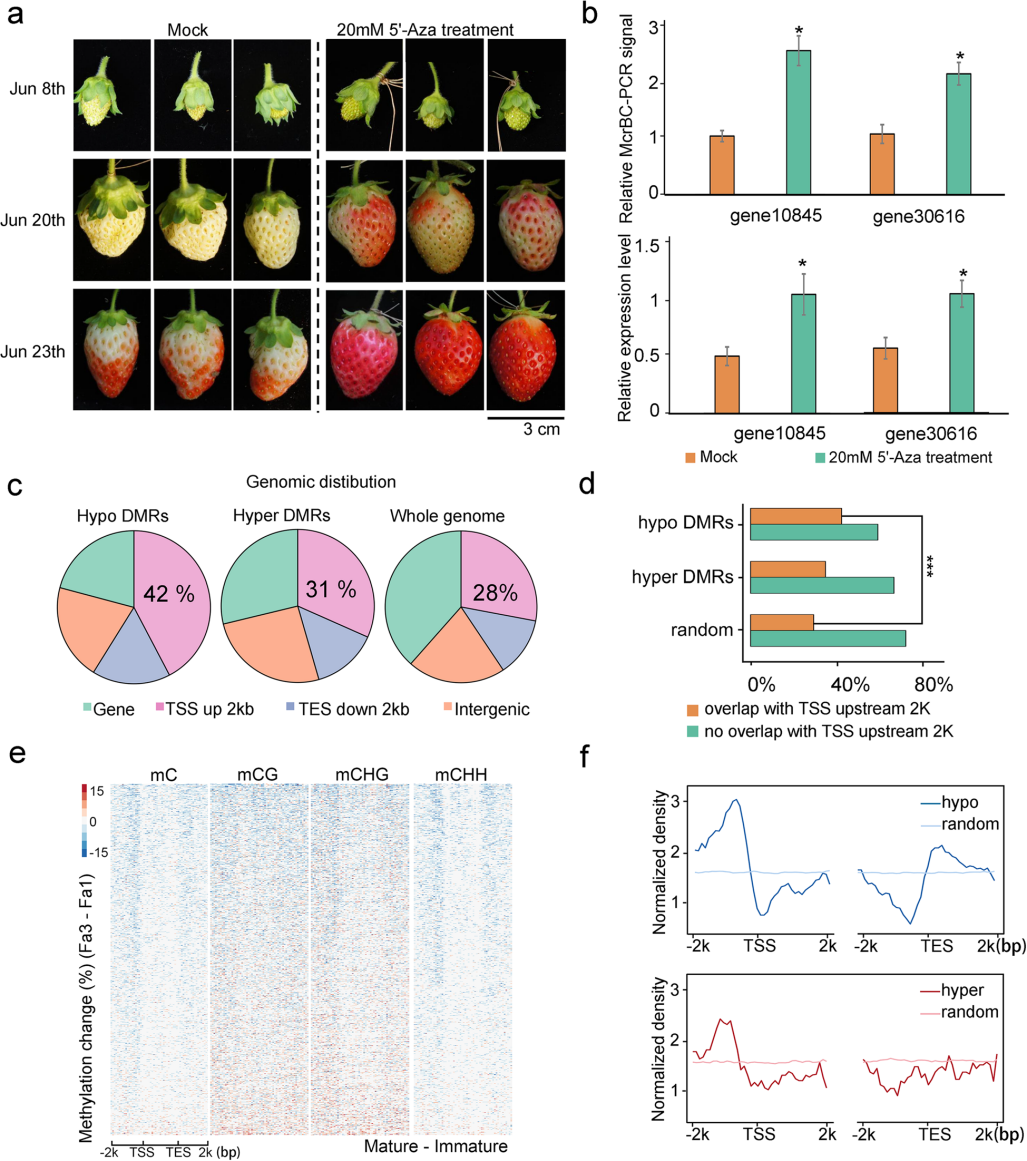
Characterization of ripening-induced DNA hypomethylation. **a** Pictures of DNA methylation inhibitor 5-azacytidine-treated fruits. Fruits treated with water served as control (mock). **b** McrBC-qPCR analysis of DNA methylation levels (upper panel) and qPCR analysis of gene expression levels (lower panel) of two genes in mock and 5-azacytidine-treated fruits. In McrBC-qPCR analysis, a higher qPCR signal indicates lower mC level. Error bars indicate SD, *n* = 3 (*P value < 0.05, two-tailed *t* test). **c** Distribution of genomic elements within ripening-induced hypo-DMRs, hyper-DMRs, and the whole genome. Gene, gene body; TSS up 2 kb, 2 kb upstream of TSS; TES down 2 kb, 2 kb downstream of TES; Intergenic, intergenic regions. **d** Hypo-DMRs are significantly associated with regions 2 kb upstream of TSS as compared to random genomic regions (***P value < 0.01, as determined using Fisher’s exact test). **e** Heatmaps showing DNA methylation changes (Fa3-Fa1) across hypo-DMR-associated genes. Methylation changes in mC, mCG, mCHG, and mCHH contexts are shown. **f** Distributions of hypo-DMRs (blue line in upper panel) and hyper-DMRs (red line in lower panel) around genes as compared to that of randomly selected genomic regions (light-blue line and light-red line)

To investigate the importance of the decreased DNA methylation during ripening, we treated young fruits with a DNA methylation inhibitor, 5-azacytidine. As shown in Fig. 3a, compared with mock treatment, 5-azacytidine-treated fruits exhibited an early ripening phenotype. To examine whether the 5-azacytidine treatment influenced fruit ripening through altering DNA methylation level in the fruits, we performed methylation-sensitive qPCR for both mock- and 5-azacytidine-treated fruits. At the two examined genomic regions, 5-azacytidine-treated fruits showed decreased DNA methylation levels compared to mock-treated fruits (Fig. 3b). These results suggest that the DNA hypomethylation is important for strawberry fruit ripening.

Next, we evaluated the genomic distribution of DMRs. We found that hypo-DMRs were enriched at genomic regions encompassing transcriptional start sites (TSS) (Fig. 3c, d). In particular, the distribution of DMRs around genes revealed that hypo-DMRs, especially at mCHH, were enriched at the 5′- and 3′-flanking regions of genes (Fig. 3e, f). In contrast, the distribution of hyper-DMRs did not show such a pattern (Fig. 3f, Additional file 2: Figure S3). These results suggest that ripening-induced DNA hypomethylation potentially regulates gene expression.

### Reduced expression of RdDM pathway genes during ripening

The DNA hypomethylation during tomato fruit ripening is due to increased expression of the DNA demethylase gene, *SlDML2* [14]. To investigate the mechanism of DNA hypomethylation during strawberry ripening, we first examined the expression of DNA demethylase genes. We generated genome-wide transcript profiles for strawberry fruit at Fa1–Fa3 stages shown in Fig. 1a, with two biological replicates at each stage that showed good consistency via PCA (Additional file 2: Figure S4a). In *Arabidopsis*, DNA demethylation is initiated by the REPRESSOR OF SILENCING (ROS1) family of glycosylase/lyase proteins, which can remove the mC base [1]. The strawberry genome harbors 4 *ROS1* homologs, including gene01635 (*FvDME1*), gene30143 (*FvROS1.1*), gene30462 (*FvROS1.2*), and gene11785 (*FvROS1.3*) (Additional file 2: Figure S4b). None of these *ROS1* homologs showed significantly increased expression during ripening (Additional file 2: Figure S4c). The IDM (Increase of DNA methylation) protein complex can regulate ROS1 targeting and, in turn, DNA demethylation in *Arabidopsis* [22, 23]. We examined the expression of strawberry genes that are similar to the *AtIDMs*, including gene27227 (*FvIDM1*), gene21977 (*FvIDM2*), and gene31466 (*FvIDM3*) (Additional file 2: Figure S4b). However, none of these was upregulated during ripening (Additional file 2: Figure S4c). These results suggest that DNA hypomethylation during strawberry ripening is not associated with increased expression of DNA demethylation pathway genes.

**Fig. 4:**
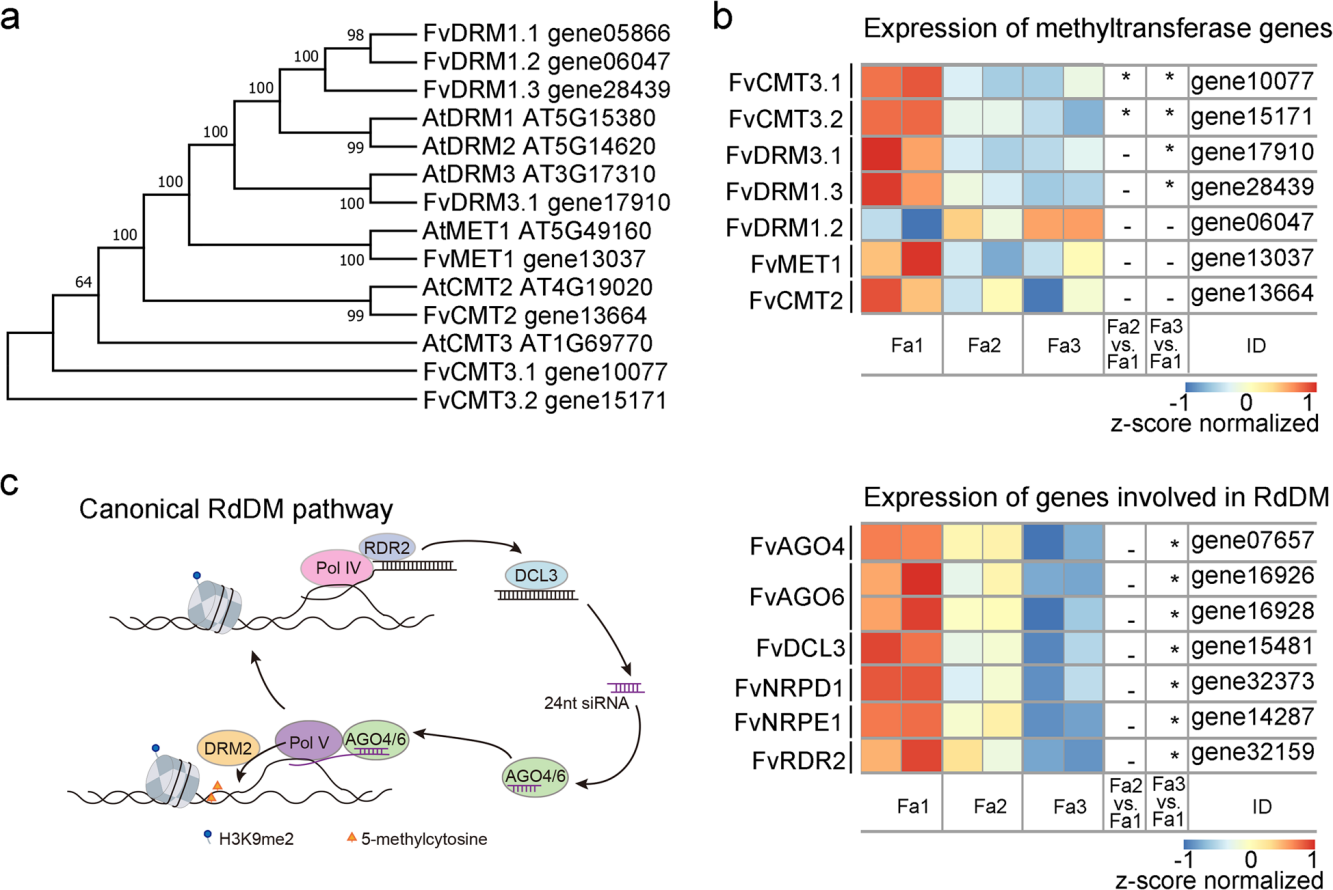
Expression of genes involved in DNA methylation. **a** Phylogenetic analysis of DNA methyltransferase genes in strawberry and *Arabidopsis*. **b** Heatmap showing transcript levels of DNA methyltransferase genes in Fa1~Fa3 (*adjusted *P* value < 0.05, as determined using the DESeq). **c** Transcript levels of genes involved in the RdDM pathway in Fa1~Fa3. A brief working model of RdDM pathway is shown on the left. Pol IV (RNA polymerase IV), RDR2 (RNA-Dependent RNA polymerase 2), and DCLs (DICER-LIKES) are required for siRNA biogenesis; Pol V, AGO4/6 (ARGONAUTE 4/6), and DRM2 are involved in siRNA-guided DNA methylation. NRPD1 and NRPE1 are the largest subunits of Pol IV and Pol V respectively. Heatmap (right panel) shows transcript levels of genes involved in RdDM pathways (* adjusted *P* value < 0.05, as determined using the DESeq)

DNA demethylation and DNA methylation can antagonize each other to dynamically regulate the plant methylome. We hypothesized that a reduced DNA methylation activity could contribute to the DNA hypomethylation during strawberry fruit ripening. To test this hypothesis, we examined the expression of genes involved in DNA methylation pathways. In *Arabidopsis*, mCG, mCHG, and mCHH are maintained by METHYLTRANSFERASE 1 (MET1), CHROMOMETHYLASE 3 (CMT3), and CMT2 and DOMAINS REARRANGED METHYLASEs (DRMs), respectively, whereas all three contexts can be de novo methylated by DRMs via the RNA-directed DNA methylation (RdDM) pathway [1, 2]. We identified 8 DNA methyltransferase genes in the strawberry genome (annotated in Phytozome), including orthologs to *AtMET1* (gene13037 (*FvMET1*)), *AtCMT*s (gene13664 (*FvCMT2*), gene10077 (*FvCMT3.1*), and gene15171 (*FvCMT3.2*)), and *AtDRM*s (gene05866 (*FvDRM1.1*), gene06047 (*FvDRM1.2*), gene28439 (*FvDRM1.3*) and gene17910 (*FvDRM3.1*)) (Fig. 4a). We found that *FvDRM1.1* was barely expressed in fruits (FPKM < 2) (data not shown) and *FvMET1*, *FvCMT2*, and *FvDRM1.2* were not differentially expressed during ripening (adjusted *P* value < 0.05) (Fig. 4b, Additional file 4: Table S3). However, *FvCMT3.1*, *FvCMT3.2*, *FvDRM1.3*, and *FvDRM3.1* were all downregulated during ripening (Fig. 4b).

The downregulation of *FvDRM1.3* and *FvDRM3.1* suggested that RdDM activity may be reduced during strawberry ripening. To further investigate whether RdDM activity is reduced during strawberry ripening, we examined the expression of other RdDM components. RdDM involves two steps, siRNA biogenesis, which requires Pol IV, RDR2, and DCL3, and siRNA-guided DNA methylation, which requires Pol V, AGO4/6, and DRMs [5] (Fig. 4c). We identified strawberry orthologs of Pol IV and Pol V largest subunits, *RDR2*, *DCL3*, *AGO4*, and *AGO6*, and found that all of these genes displayed reduced transcript levels from Fa1 to Fa3 (adjusted *P* value < 0.05). The expression patterns of these genes were confirmed by analysis of published transcriptome data (see methods for detailed information) (Additional file 2: Figure S4d). The decreased expression of *FvCMTs* as well as *FvDRMs* and other RdDM pathway genes is consistent with the observed loss of DNA methylation during ripening, especially the loss of non-CG methylation. Together, these data suggest that reduced DNA methylation activities contribute to ripening-induced DNA hypomethylation.

### siRNAs are diminished at ripening-induced hypo-DMRs

The decreased expression of genes involved in RdDM during ripening (Fig. 4) suggests that RdDM activity is reduced. To assess RdDM activity more directly, we examined the levels of RdDM-dependent 24-nt siRNAs [2].

**Fig. 5:**
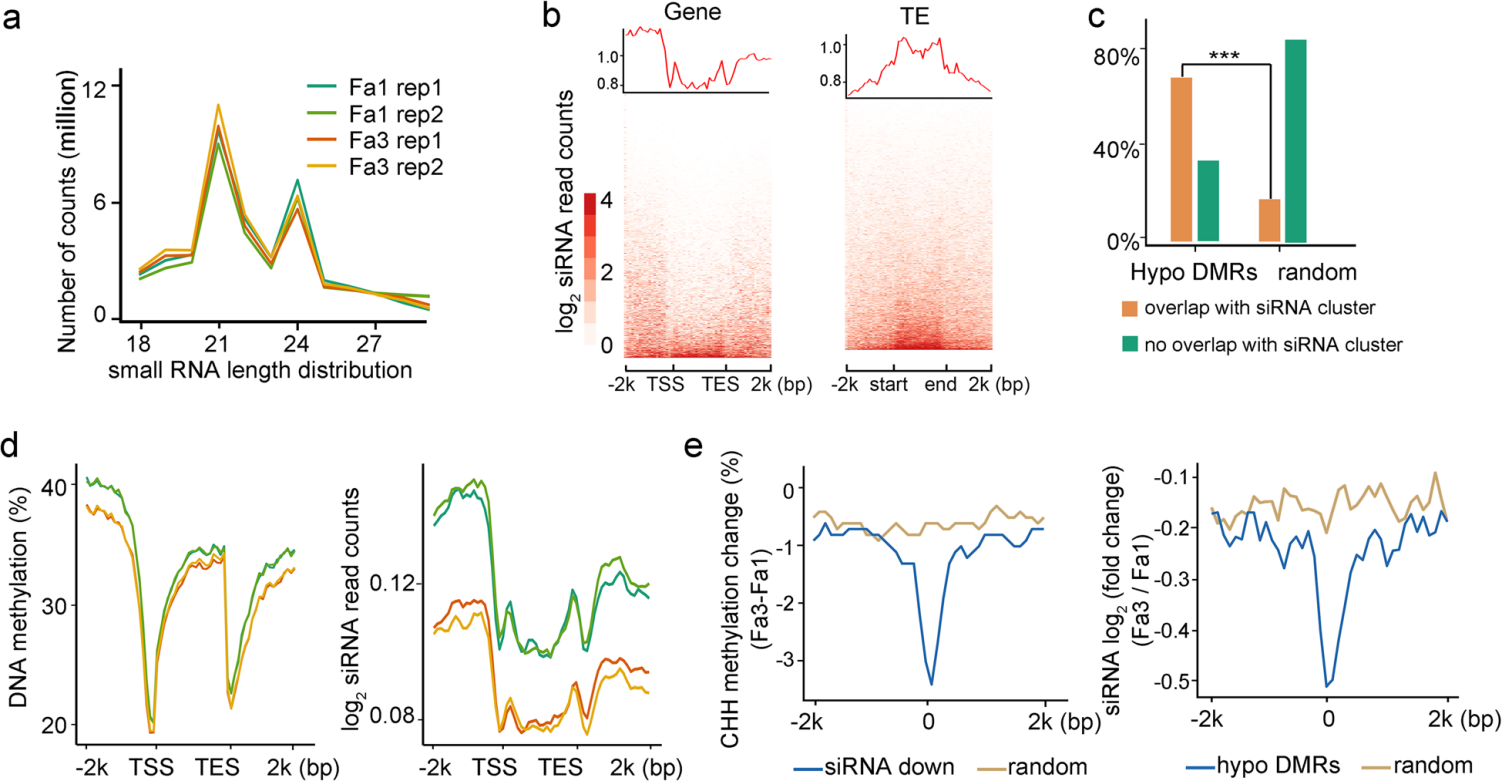
Association between ripening-induced siRNA decrease and DNA hypomethylation. **a** Size distribution of sequenced strawberry small RNAs. Two biological replicates of Fa1 and Fa3 are shown. **b** Average profile and heatmaps showing 24-nt siRNA distribution surrounding genes (left) and TEs (right). **c** Hypo-DMRs are significantly associated with siRNA clusters as compared to random genomic regions (***P value < 0.01, as determined using Fisher’s exact test). **d** Profiles of DNA methylation (left panel) and siRNA (right panel) surrounding genes in Fa1 and Fa3. Two biological replicates of Fa1 and Fa3 are shown. **e** Change of CHH methylation surrounding siRNA downregulated regions (left) and change of 24-nt siRNA level surrounding hypo-DMRs (right) on average

First, we used deep sequencing to evaluate the genome-wide siRNA profiles in Fa1 and Fa3. Consistent with the small RNA composition in *Arabidopsis*, 21-nt and 24-nt small RNAs represented the most abundant small RNAs in all sequenced samples of strawberry fruits (Fig. 5a). RdDM-dependent 24-nt siRNAs in *Arabidopsis* are characterized by an adenosine at the 5′-end [24]. We found that 24-nt siRNAs in strawberry also have a preference for 5′ terminal adenosine (Additional file 2: Figure S5a), suggesting that 24-nt siRNA biogenesis is conserved in strawberry and *Arabidopsis*. Further analysis revealed that strawberry 24-nt siRNAs are preferentially located in promoter and 3′ regulatory regions around genes, but are more widely spread along TE bodies and flanking regions (Fig. 5b).

The biogenesis of 24-nt siRNA depends on RdDM. However, in *Arabidopsis*, it is known that long TEs usually have siRNAs in their body regions, even though their body methylation relies on CMT2 [3]. In strawberry, we identified 370,630 24-nt siRNA clusters (Additional file 5: Table S4); 345,828 of these clusters are not located in long TE bodies and thus can be considered as canonical RdDM target regions. We found that over 67% of ripening-induced hypo-DMRs overlapped with the siRNA clusters (Fig. 5c). In contrast, only 17% of randomly selected genomic regions overlapped with the siRNA clusters, suggesting that ripening-induced hypo-DMRs are significantly associated with siRNAs (*p* < 0.01) (Fig. 5c). Further, ripening led to diminished DNA methylation and decreased numbers of siRNA clusters in the bodies and flanking regions of genes and TEs (Fig. 5d, Additional file 2: Figure S5b). To further study the relationship between siRNA-mediated DNA methylation and ripening-induced DNA hypomethylation, we monitored changes in siRNA enrichment at ripening-induced hypo-DMRs. We found that siRNA levels were reduced at ripening-induced hypo-DMRs from Fa1 to Fa3 (Fig. 5e and Additional file 2: Figure S5c). In addition, we observed a decreased mCHH level at genomic regions with reduced siRNA levels (Fig. 5e). The siRNA levels at several representative hypo-DMRs are shown in Fig. 2b. To test the significance of RdDM pathway on strawberry ripening, we used TRV-mediated gene silencing to downregulate *FvAGO4* in young strawberry fruits. We observed an early ripening phenotype in *TRV2:FvAGO4* fruits compared to control TRV2-only fruits (Additional file 2: Figure S5d), consistent with the involvement of RdDM in strawberry ripening. These analyses suggest that the reduced expression of RdDM pathway genes leads to diminished siRNA accumulation and decreased RdDM activity, thus contributing to DNA hypomethylation and ripening of strawberries.

### DNA methylation changes are associated with altered gene expression in fruits

**Fig. 6:**
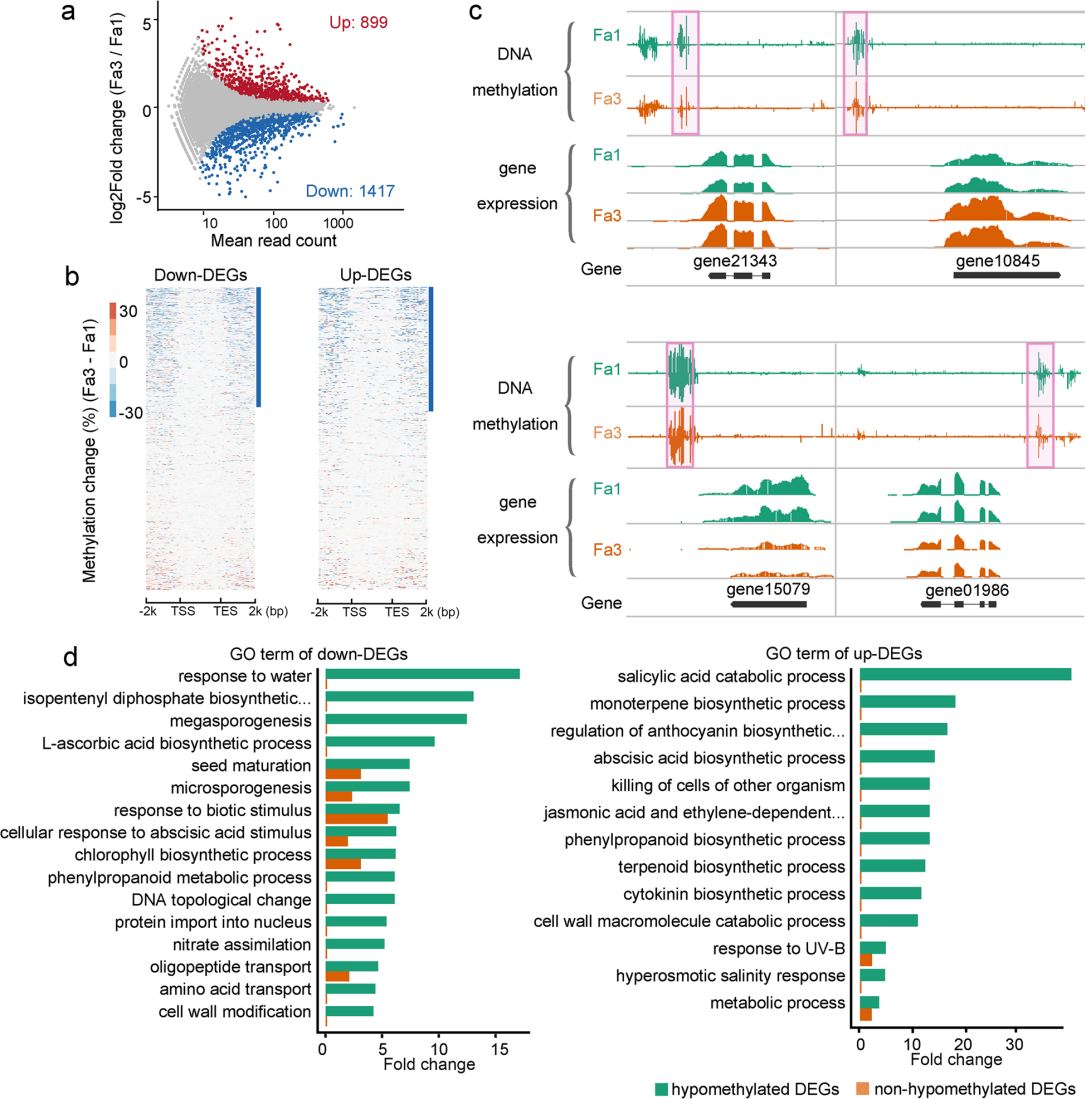
Association between DNA methylation and gene expression changes. **a** MA-plot showing the expression change during ripening. Eight hundred ninety-nine up- and 1417 down-differentially expressed genes (DEGs) were detected in Fa3 as compared to Fa1 (adjusted *P* value < 0.01, as determined using the DESeq). **b** Heatmaps showing DNA methylation changes (Fa3-Fa1) surrounding 1417 down-DEGs (left panel) and 899 up-DEGs (right panel). The blue bars on the right indicate hypomethylated genes. **c** IGB display of DNA methylation levels and transcript levels of two up-DEGs (upper panel) and two down-DEGs (lower panel) in Fa1 and Fa3. Hypo-DMRs are boxed. **d** Gene Ontology (GO) analysis of hypomethylated DEGs and non-hypomethylated DEGs. GO terms enriched in hypomethylated-DEGs were illustrated. GO enrichments of hypomethylated down-DEGs (left panel) and up-DEGs (right panel) are shown respectively

To investigate the relationship between ripening-induced changes in DNA methylation and gene expression, we compared the transcriptomes for fruits at Fa1 and Fa3. We identified a total of 2316 differentially expressed genes (DEGs, adjusted *P* value < 0.01), including 899 upregulated DEGs (up-DEGs) and 1417 downregulated DEGs (down-DEGs) in Fa3 relative to Fa1 (Fig. 6a and Additional file 6: Table S5). Next, we examined the DNA methylation levels at the up- and downregulated DEGs. We found that a large subset of the DEGs undergoes a loss of DNA methylation from Fa1 to Fa3, especially in non-CG contexts, at their 5′- and 3′-regulatory regions (Fig. 6b-c, Additional file 2: Figure S6a and Additional file 6: Table S5). DNA methylation, especially at promoters, is often associated with transcriptional silencing. For upregulated DEGs, the decrease in DNA methylation corresponded to increased expression during ripening, which is consistent with the role of DNA methylation in gene silencing (Fig. 6c). To investigate the significance of DNA methylation in the regulation of gene expression, we examined the methylation and expression levels of two up-DEGs (gene10875 and gene30616) in mock and 5-azacytidine-treated samples. Compared with mock treatment, the promoter regions of these two genes were hypomethylated and their expression was upregulated in 5-azacytidine-treated fruits, suggesting a repressive role of DNA methylation for these two genes (Fig. 3b). For downregulated DEGs, the DNA hypomethylation corresponded to decreased expression during ripening (Fig. 6b, c). A recent study using tomato DNA demethylase mutants revealed that DNA demethylation is associated with the repression of several hundred genes during tomato ripening [14]. How DNA demethylation causes gene repression is still unclear in both tomato and strawberry.

Fruit ripening is a complex developmental process that involves numerous physiological, biochemical, and structural alterations. To understand how DNA methylation-associated gene regulation contributes to the ripening process, we performed Gene Ontology (GO) analysis for hypomethylated up- and downregulated DEGs. Up- and downregulated DEGs that are not hypomethylated served as controls (Fig. 6d). This analysis revealed that genes involved in the “regulation of anthocyanin biosynthetic process” are enriched in upregulated DEGs (Fig. 6d and Additional file 7: Table S6), suggesting that DNA methylation may regulate fruit coloration during ripening. A previous study found that gene21343 (expansin-A8-like) is required for biosynthesis of the pigment anthocyanin during strawberry ripening [25], although the underlying molecular mechanism was unclear. Our results showed that gene21343 displayed decreased DNA methylation during ripening, and its expression was upregulated accordingly (Fig. 6b, Additional file 2: Figure S6b). Cytokinin is important for early fruit development, and a recent study suggested that cytokinin accumulates during the ripening of kiwi, grape, and strawberry fruits [26]. Consistent with these findings, our analysis found that genes involved in the “cytokinin biosynthetic process” are enriched in the upregulated DEGs (Fig. 6d and Additional file 7: Table S6). The endogenous content of ABA is known to increase substantially during strawberry ripening, and the application of an ABA synthesis inhibitor interrupts strawberry ripening [8]. Consistent with these observations, we found that genes involved in the “abscisic acid biosynthetic process” are enriched in the upregulated DEGs (Fig. 6d and Additional file 7: Table S6). Several other ripening-related GO terms were also included in the upregulated DEGs, such as genes involved in the biosynthesis of flavor volatiles (Fig. 6d and Additional file 7: Table S6).

On the other hand, many genes need to be downregulated during ripening. For example, genes involved in photosynthesis are active and required in the early stages of fruit development, but are repressed during ripening to allow conversion of chloroplasts into chromoplasts, which are important for fruit coloration. The hypomethylated downregulated DEGs were enriched for “chlorophyll biosynthetic process,” suggesting that DNA hypomethylation may contribute to the decline in photosynthesis during ripening. We also found several GO terms related to cell wall biosynthesis and metabolism in hypomethylated downregulated DEGs. During early fruit development, genes involved in cell wall biosynthesis and modification are active to sustain fast cell division and cell enlargement, but these genes are not needed later during the ripening stage. Interestingly, the ripening-related GO terms were only enriched in hypomethylated DEGs, but not enriched in up- or down-DEGs without hypomethylation (Fig. 6d and Additional file 7: Table S6). Together, these analyses suggest that DNA methylation-mediated gene regulation controls many biological processes important for ripening.

## Discussion

DNA methylation was proposed to be an important factor in the regulation of fruit ripening, mainly based on studies in tomatoes [7]. Tomato, a typical climacteric fruit, undergoes genome-wide loss of DNA methylation during ripening [12]. However, the dynamics and relevance of DNA methylation in non-climacteric fruit ripening, or in any other climacteric fruit, were unknown. Here, we generated single-base resolution maps of DNA methylation for strawberry leaves and fruits at different stages (Fig. 1a). We found that, similar to tomatoes, strawberry fruits undergo an overall loss of DNA methylation, especially in non-CG contexts, during ripening (Fig. 2) [14]. Our results suggest that ripening-induced DNA hypomethylation could occur in both climacteric and non-climacteric fruits.

DNA methylation can be dynamically regulated by DNA methylation and DNA demethylation activities. In *Arabidopsis*, active DNA demethylation is catalyzed by ROS1 family proteins, which can remove the mC base and cleave the DNA backbone, leaving a single-nucleotide gap that can be filled with a non-methylated cytosine [1]. The loss of DNA methylation during tomato fruit ripening is caused by increased expression of *SlDML2*, a tomato ortholog of *Arabidopsis ROS1* [14]*.* However, none of the DNA demethylase genes in strawberry was upregulated during ripening (Additional file 2: Figure S4). Instead, we found that several genes encoding DNA methyltransferases and other key components in the RdDM pathway were significantly downregulated during strawberry fruit ripening (Fig. 4). We inferred that RdDM activity is reduced during strawberry ripening, such that DNA demethylation becomes relatively dominant over methylation, leading to an overall loss of DNA methylation. In our model, even though DNA methylation activities are decreasing, the active DNA demethylation machinery must be active during ripening, to remove the existing DNA methylation, since there is little cell division in ripening fruits and thus passive demethylation should be insignificant. Indeed, we found that two of the strawberry DNA demethylase genes were expressed during ripening (FPKM > 20).

RdDM is a well-known DNA methylation pathway that consists of two main steps: siRNA biogenesis and siRNA-guided DNA methylation. The enrichment of 24-nt siRNAs is an important indicator of RdDM activity. We generated and characterized genome-wide siRNA profiles for immature and ripe strawberry fruits (Fig. 5) and found that the number of siRNA clusters decreased during ripening. Further, the genomic regions that displayed reduced DNA methylation (hypo-DMRs) also displayed decreased siRNA enrichment during ripening (Fig. 5). Our siRNA analysis provides strong evidence for the diminished RdDM activity during strawberry ripening and also further supports our model that weakened RdDM contributes to the ripening-induced DNA hypomethylation. RdDM has been extensively studied in *Arabidopsis*; however, a regulatory role for RdDM in plant development has not been established. Here, our study suggests a novel function of RdDM in the regulation of non-climacteric fruit ripening. In addition, strawberry *CMT3* was downregulated during strawberry ripening, which could also contribute to the observed DNA hypomethylation.

We found that DNA hypomethylation and siRNA reduction preferentially occurred in the 5′- and 3′-regulatory regions of genes (Fig. 5d), suggesting that RdDM-mediated DNA methylation regulates gene expression during strawberry ripening. Consistent with this hypothesis, genes involved in ripening-related processes, such as photosynthesis, cell wall biosynthesis, ABA biosynthesis, and fruit coloration, showed altered expression and DNA hypomethylation in their promoters during ripening. Interestingly, DNA hypomethylation did not always correlate with gene activation, but was also associated with gene repression during strawberry ripening. This is especially evident for photosynthesis and cell wall synthesis-related genes, which are no longer needed in ripe fruits and must be repressed so that they do not interfere with ripening. Ripening-associated DNA hypomethylation and gene repression were also observed in tomato, where mutations in the DNA demethylase SlDML2 prevented the DNA hypomethylation and consequently the repression of photosynthesis and cell wall synthesis-related genes was obstructed [14]. How promoter DNA methylation may help activate genes is not known. However, it is clear that although many transcription factors are sensitive to DNA methylation, some actually prefer binding to methylated motifs [27] and thus may activate the methylated genes. In addition, it is possible that the DNA binding of some transcriptional repressors may be sensitive to DNA methylation, and thus, loss of methylation would allow the repressors to reduce gene expression.

## Conclusions

Our work suggests that DNA hypomethylation regulates the ripening of the non-climacteric strawberry fruit, as it does in the climacteric tomato fruit. Distinct mechanisms underlie the reduced levels of DNA methylation during strawberry and tomato ripening: diminished RdDM-mediated methylation versus increased DNA demethylation, respectively. DNA hypomethylation during ripening is associated with the altered expression of hundreds of ripening-related genes, suggesting that RdDM regulation of a genetic program contributes to fruit ripening.

## Methods

### Plant materials

Strawberries (*Fragaria × ananassa* Duch. cv. Hongjia) were grown in the scientific research innovation base at Zhejiang Academy of Agricultural Science at Haining (Zhejiang, China). Fruits at three developmental stages (Fa1, green stage, approximately 20 days after bloom (DAB); Fa2, intermediate red stage, 30 DAB; Fa3, full red stage, 35 DAB) were harvested (Fig. 1a). After being transferred to the lab on the day of harvest, fruits with uniform size and free of visible defects were selected. Two biological replicates of 8–12 fruits each were used for each developmental stage.

For DNA methylation inhibitor treatment, “Si gongzhu” strawberry fruits were used. In the treatment, 20 mM 5-azacytidine (Sigma) dissolved in ddH_2_0 with 0.01% Triton X-100 was directly sprayed on the fruits. The treatment was performed on Jun. 8, 13, and 18. The samples were pictured on Jun. 23.

### Methylation-sensitive PCR

Genomic DNA (100 ng) was digested with McrBC for 12 h according to the manufacturer’s instructions. Digestion without GTP was used as negative control. After enzyme inactivation at 65 °C for 20 min, 10% of digested DNA was used for each PCR reaction for Quantitative Real-time PCR. The primer sequence is included in Additional file 8: Table S7.

### Whole genome bisulfite sequencing and data analysis

Genomic DNA was extracted from leaves and fruits using a DNeasy Plant Maxi Kit (Qiagen). The samples were sequenced at the Genomics Core Facility of the Shanghai Centre for Plant Stress Biology, Chinese Academy of Sciences, using Illumina HiSeq2500. The libraries of whole-genome bisulfite sequencing were prepared using NEBNext Ultra II DNA Library Prep Kit for Illumina (New England Biolabs) and Epitect Plus DNA Bisulfite Kit (Qiagen). Briefly, 1 μg of genomic DNA was sonicated into 200~500 bp fragments on a Covaris M220. The fragmented DNA was subjected to end repair, A-tailing, and adaptor ligation following the manufacturer’s instructions of the NEBNext Ultra II DNA Library Prep Kit. The adapter-ligated products were then treated with sodium bisulfite on a thermocycler using Epitect Plus DNA Bisulfite Kit (Qiagen) with the following program: 95 °C 5 min, 60 °C 25 min, 95 °C 5 min, 60 °C 85 min, 95 °C 5 min, 60 °C 175 min, and 3× (95 °C 5 min, 60 °C 180 min), hold at 20 °C. The BS-treated DNA was then cleaned up using Epitect Plus DNA Bisulfite Kit (Qiagen) and PCR-amplified using the KAPA HiFi Hotstart ReadyMix for 6 cycles. The amplified libraries were finally cleaned up using magnetic beads from Vazyme. The libraries were sequenced on a HiSeq2500 (Illumina) in paired-end 125 bp mode using the HiSeq PE Cluster Kit v4 (Illumina) and HiSeq SBS Kit v4 (250 cycles) (Illumina) following the manufacturer’s instructions at Core Facility for Genomics of Shanghai Center for Plant Stress Biology.

For data analysis, paired-end sequencing reads were first trimmed with Trimmomatic [28] for removal of Illumina adapters and low-quality bases (quality score < 20). The cleaned reads were then aligned to *Fragaria vesca* genome (*Fragaria vesca v1.1*, https://phytozome.jgi.doe.gov/pz/portal.html#!info?alias=Org_Fvesca) using bsmap-2.87 [29] with default settings. Methylation ratio was extracted with methratio.py (a script included in bsmap-2.87). Properly mapped paired-end reads whose SAM flags equal 83 and 163, or 99 and 147, were used by setting options -u and -p in methratio.py. DMCs were defined using Fisher’s exact test (*p* < 0.05). DMRs were identified as previously described [30] with minor modifications. In brief, only cytosines with a depth of at least four in all libraries were considered. A sliding-window approach with window size 200-bp and step size 50-bp was used to identify DMRs. Fisher’s exact test was performed for methylated vs. unmethylated cytosines within each window. False discovery rates (FDRs) were estimated using a Benjamini-Hochberg adjusted *P* value. Windows with FDR < 0.05 were defined as candidates for further analysis. DMRs were then adjusted by merging the candidates in all three contexts and shrinking to the first and last differentially methylated cytosines (DMCs). Final DMRs were filtered with combined criteria: number of DMC > 3 and mean methylation difference > 0.15. To incorporate biological replicates into analysis, we calculated robust index as the formula for each DMR to measure the repeatability between replicates.

$$ \mathrm{Robust}\kern0.5em \mathrm{index}=\frac{\left|{\log}_2{FC}_1-{\log}_2{FC}_2\right|}{\left|{\log}_2{FC}_1+{\log}_2{FC}_2\right|} $$

FC_1_ and FC_2_ represent differential methylation fold change in replicate 1 and replicate 2, respectively. DMRs with lower robust index are more credible in terms of differential methylation. Robust index for DMRs with low depth in individual replicates were assigned as NA. We ranked the DMRs by robust index (Additional file 3: Table S2) and re-analyzed both top 500 and least 500 credible hypo DMRs respectively. As shown in Additional file 2: Figure S7, the analysis of both groups of DMRs supports major conclusions in the main text, including gradual decrease in DNA methylation during ripening (Additional file 2: Figure S7a), enrichment of DMRs in promoter regions (Additional file 2: Figure S7b), and decreased siRNA accumulation in hypo-DMRs (Additional file 2: Figure S7c). Principal component analysis (PCA) was used to cluster the methylation patterns of fruits and leaves in DMRs into low dimensions. Integrated Genome Browser (IGB) [31] was used to visualize the DNA methylation data. DMR-associated genes were defined as genes with the closest DMR located within 2 kb upstream of the transcription start site (TSS) and 2 kb downstream of the transcription end site (TES)).

### RNA sequencing and data analysis

Total RNA was extracted with TRIzol reagent (Ambion) from fruits. For reverse transcription, 1 μg of RNA and oligo dT primers were used to synthesize cDNA in a 20-μL reaction using the qScript cDNA SuperMix kit (Quanta). For RNA-seq, the libraries were constructed and sequenced at the Genomics Core Facility of the Shanghai Centre for Plant Stress Biology, Chinese Academy of Sciences, using an Illumina HiSeq2500.

For data analysis, paired-end reads were aligned to *Fragaria vesca* genome (*Fragaria vesca v1.1*, https://phytozome.jgi.doe.gov/pz/portal.html#!info?alias=Org_Fvesca) using STAR [32] with default parameters. Uniquely mapped reads with MAPQ > 20 were collected for further analysis. FeatureCounts [33] was used to count the mapped fragments for each gene. DESeq [34] was used to detect differentially expressed genes. To assign putative functions of genes in *Fragaria vesca*, gene orthologs provided by Phytozome [35] (https://phytozome.jgi.doe.gov/pz/portal.html) were collected and the function of corresponding *Arabidopsis* ortholog was assigned. Fisher’s exact test was used to determine whether the gene set is significantly enriched in a specific GO term. Enriched GO terms were defined base on combined criteria: |log_2_ Fold change| > 1 and *P* value < 0.05. To valid the gene expression changes during ripening, published datasets (accession number PRJNA394190 (https://www.ncbi.nlm.nih.gov/bioproject/394190)) which characterized the transcriptome change during strawberry ripening were downloaded and processed.

### Small-RNA sequencing and data analysis

Total RNA was extracted with TRIzol reagent (Ambion) from fruits. Total RNA was then separated on denaturing polyacrylamide gels, and < 100-nt fractions were cut out and purified for standard small RNA library preparation. For small-RNA sequencing, the libraries were constructed and sequenced at the Genomics Core Facility of the Shanghai Centre for Plant Stress Biology, Chinese Academy of Sciences, using an Illumina HiSeq2500.

Sequenced reads were trimmed with Trimmomatic [28] to remove Illumina adapters and low-quality bases (quality score < 20). Reads with length < 18 bp and length > 30 bp were discarded. Cleaned 24-nt reads were mapped to *F. vesca* genome and defined to siRNA cluster with Shortstack [36]. The mapped reads were normalized by total cleaned reads for further analysis.

### Phylogenetic analysis

The orthologs of DNA methyltransferase, DNA demethylase, and gene involved in RdDM pathway annotated in Phytozome were used to build a Neighbor-Joining phylogenetic trees with MEGA [37].

### Virus-induced gene silencing (VIGS)

The TRV vectors pTRV1 (pYL192) and pTRV2 (pYL156) for gene silencing have been described by Liu et al. [38], and the treatment protocol was as described by Birch-Smith et al. [39]. pTRV2 vector was ligated with PCR fragment of *FvAGO4* and then transformed into A. tumefaciens GV3101 strain. The Agrobacterium culture (grown in 25 mg/L Rifampicin and 50 mg/L Kanamycin overnight culture) was transformed with pTRV1, pTRV2 and pTRV2 derivative pTRV2-*FvAGO4*, respectively. Agrobacterium culture was infiltrated into fruit pedicel of strawberry.

## Additional files

Additional file 1: **Table S1.** Mapping statistics of bisulfite sequencing, RNA sequencing, and small-RNA sequencing. (XLSX 19 kb)

Additional file 2: **Figure S1.** The strawberry methylomes. **Figure S2** Methylomes of fruits at different stages. **Figure S3** Heatmaps showing DNA methylation changes (Fa3-Fa1) across hyper-DMR-associated genes. **Figure S4** Expression of genes involved in DNA demethylation. **Figure S5** Analyses of siRNAs in strawberry fruits. **Figure S6** Association between gene expression and DNA methylation during ripening. **Figure S7** Repeatability between replicates using robust index. (DOCX 14141 kb)

Additional file 3: **Table S2.** Summary of 466 hyper DMRs and 2300 hypo DMRs in Fa3 compared to Fa1. (XLSX 283 kb)

Additional file 4: **Table S3.** Expression of genes involved in DNA methylation and demethylation during ripening process. (XLSX 17 kb)

Additional file 5: **Table S4.** Genomic coordinates of 370,630 24-nt siRNA clusters in strawberry fruits. (XLSX 7562 kb)

Additional file 6: **Table S5.** Summary of 2316 genes with differential expression and differential methylation levels in Fa3 compared to Fa1. (XLSX 317 kb)

Additional file 7: **Table S6.** Enriched GO terms of 555 hypomethylated down-DEGs, 367 hypomethylated up-DEGs, 862 non-hypomethylated down-DEGs, and 532 non-hypomethylated up-DEGs. (XLSX 36 kb)

Additional file 8: **Table S7.** Primers for q-PCR and VIGS. (XLSX 9 kb)

Additional file 9: Review history. (DOCX 57 kb)

## Abbreviations

5′-Aza: 5-Azacytidine
ABA: Abscisic acid
AGO4/6: Argonaute 4/6
At: *Arabidopsis thaliana*
CMT2: Chromomethylase 2
CMT3: Chromomethylase 3
CNR: Colorless non-ripening
DCLs: Dicer-likes
DEG: Differentially expressed genes
DMCs: Differentially methylated cytosines
DMRs: Differentially methylated regions
DRMs: Domains rearranged methylases
FDR: False discovery rates
Fv: *Fragaria vesca*
Fa: *Fragaria x ananassa*
VIGS: *Virus-induced gene silencing*
GO: Gene ontology
IDM: Increase of DNA methylation
MET1: Methyltransferase 1
NCED: 9-cis-epoxycarotenoid dioxygenase
PCA: Principal component analysis
Pol IV: RNA polymerase IV
Pol V: RNA polymerase V
RdDM: RNA-directed DNA methylation
ROS1: Repressor of silencing 1
siRNA: Small interfering RNA
Sl: *Solanum lycopersicum*
TE: Transposable element
MAPQ: Mapping quality
TSS: Transcriptional start sites
TES: Transcriptional end sites
mC: DNA methylation of all cytosines sites
mCG: DNA methylation of CG sites
mCHG: DNA methylation of CHG sites
mCHH: DNA methylation of CHH sites
FPKM: Fragments per kilobase million
Chr: Chromosome
