# Supplementary material for: Downregulation of RdDM during strawberry fruit ripening
Source: Genome Biol. 2018 Dec 4;19:212. doi: 10.1186/s13059-018-1587-x (PMC6280534; doi:10.1186/s13059-018-1587-x)
Supplement: Supplementary file 2 — Figure S1. The strawberry methylomes. Figure S2 Methylomes of fruits at different stages. Figure S3 Heatmaps showing DNA methylation changes (Fa3-Fa1) across hyper-DMR-associated genes. Figure S4 Expression of genes involved in DNA demethylation. Figure S5 Analyses of siRNAs in strawberry fruits. Figure S6 Association between gene expression and DNA methylation during ripening. Figure S7 Repeatability between replicates using robust index. (DOCX 14141 kb) [file 13059_2018_1587_MOESM2_ESM.docx]

[Additional File2](#_Toc529457811)

[Figure S1. The strawberry methylomes. 2](#_Toc529457812)

[Figure S2. Methylomes of fruits at different stages. 3](#_Toc529457813)

[Figure S3. Heatmaps showing DNA methylation changes (Fa3-Fa1) across hyper-DMR-associated genes. 4](#_Toc529457814)

[Figure S4. Expression of genes involved in DNA demethylation. 5](#_Toc529457815)

[Figure S5. Analyses of siRNAs in strawberry fruits. 6](#_Toc529457816)

[Figure S6. Association between gene expression and DNA methylation during ripening. 7](#_Toc529457817)

[Figure S7. Repeatability between replicates using robust index. 8](#_Toc529457818)


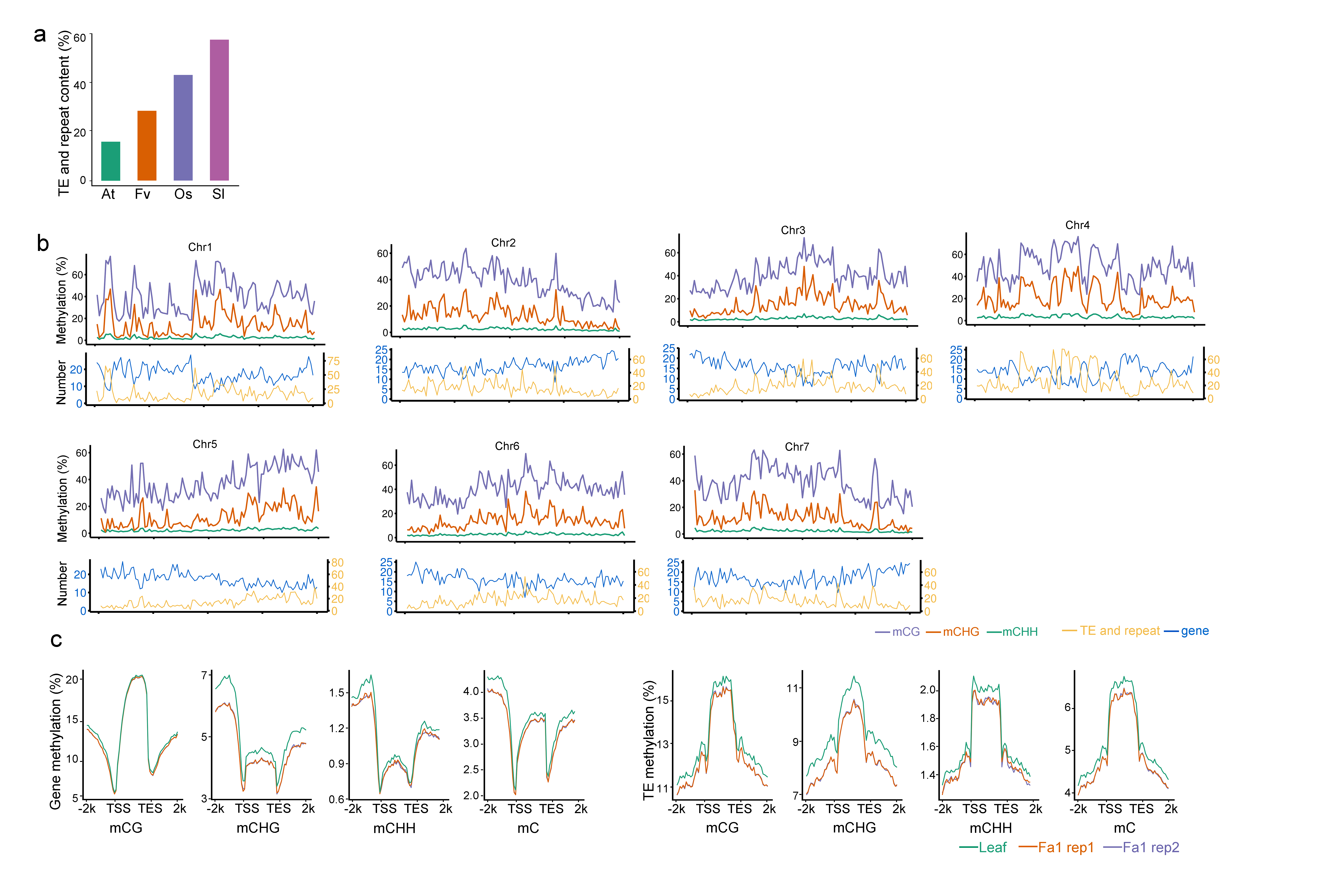


Figure S1. The strawberry methylomes.

1. TE and repeat contents in genomes of *Arabidopsis* (At), strawberry (Fv), rice (Os) and tomato (Sl).
2. Distribution of mCG, mCHG, mCHH, gene density and TE density across strawberry chromosomes (Chr1-7). Y-axis in upper panel represents the percentage of methylated DNA. Y-axis in lower panel represents density of TEs or genes normalized by the length of chromosomes.
3. DNA methylation profiles of mC, mCG, mCHG and mCHH surrounding genes (upper panel) and TEs (lower panel) in leaf and fruit tissues (Fa1). Two biological replicates of Fa1 methylome were used. Transcription start site (TSS) and transcription end site (TES) are indicated.


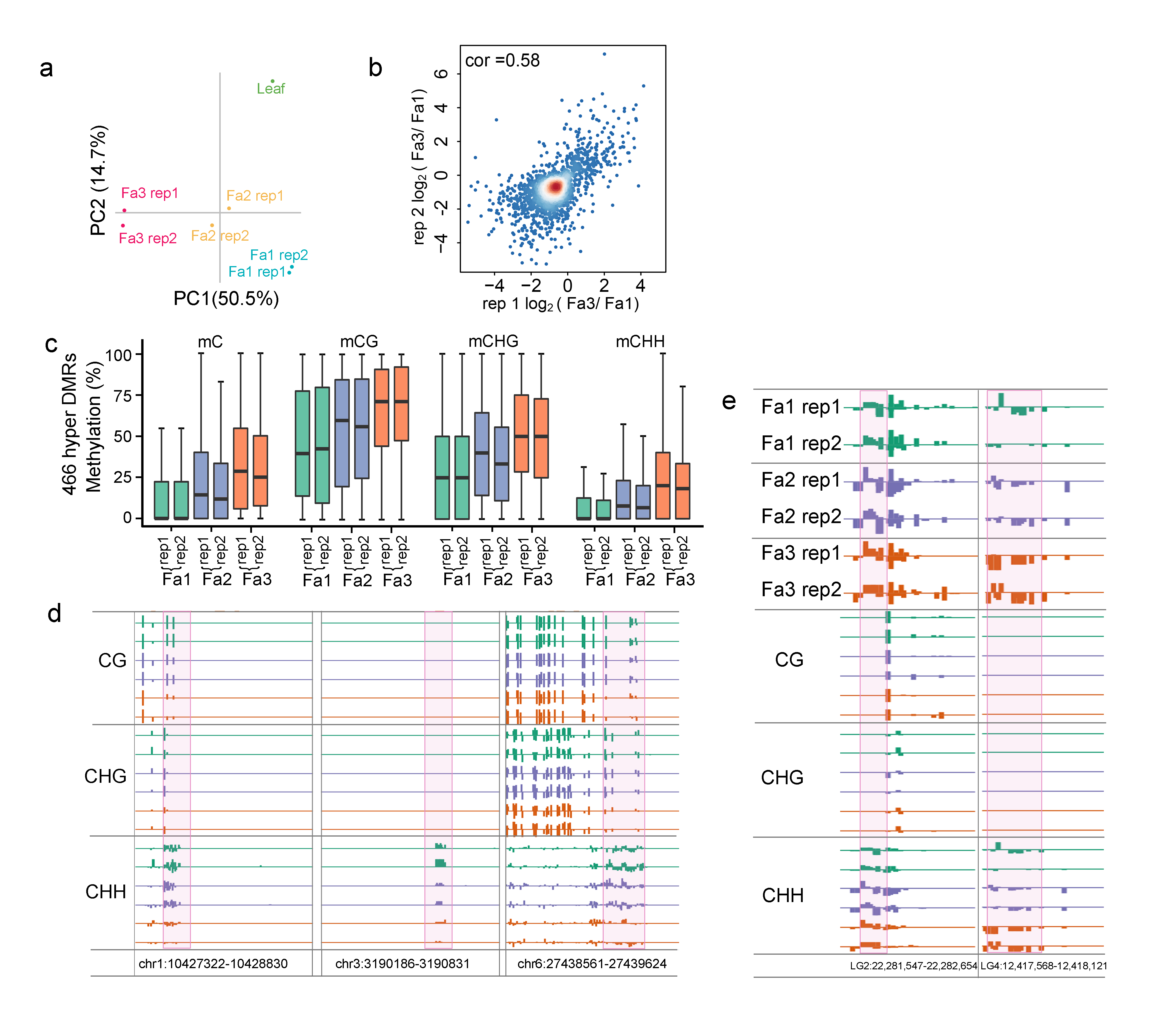


Figure S2. Methylomes of fruits at different stages.

1. Principle component analysis (PCA) showing consistency between two biological replicates of fruits at each stage. Whole genomic bisulfite-sequencing samples of leaf and Fa1~ Fa3 are shown.
2. Scatter plot showing the correlation of DNA methylation change between biological replicates
3. Boxplots showing DNA methylation levels of 466 hyper-differentially methylated regions (DMRs) in Fa3 relative to Fa1 in all stages. Methylation levels in mC, mCG, mCHG, and mCHH contexts are shown respectively. Two biological replicates were used.
4. IGB display of DNA methylation levels (CG, CHG and CHH context) of four ripening-induced hypo-DMRs. Genome coordinates are indicated in the bottom. Two biological replicates were used.
5. IGB display of DNA methylation levels of two ripening-induced hyper-DMRs. Genome coordinates are indicated in the bottom. Two biological replicates were used.


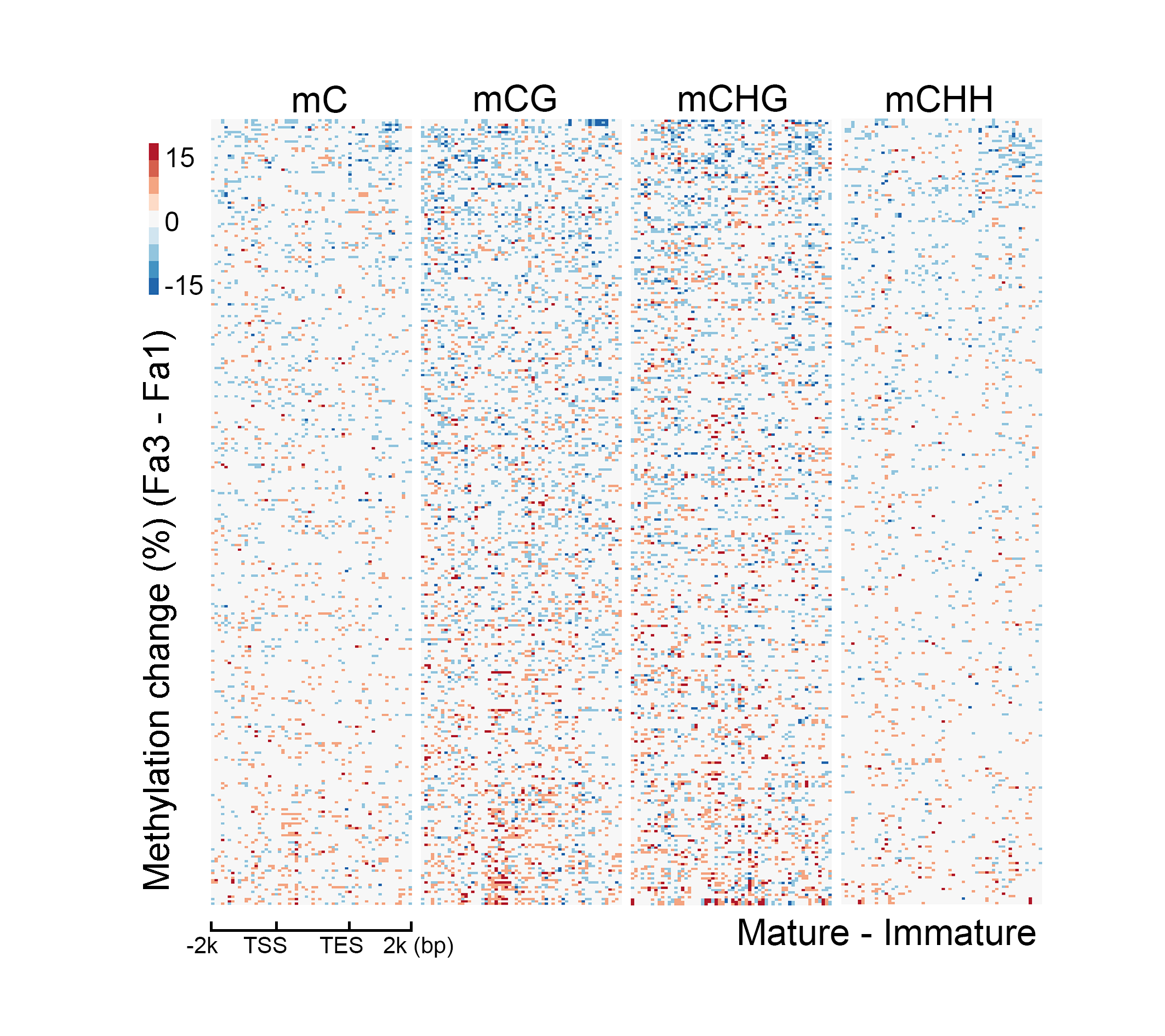


Figure S3. Heatmaps showing DNA methylation changes (Fa3-Fa1) across hyper-DMR-associated genes. DNA methylation changes are shown for mC, mCG, mCHG, and mCHH contexts respectively.


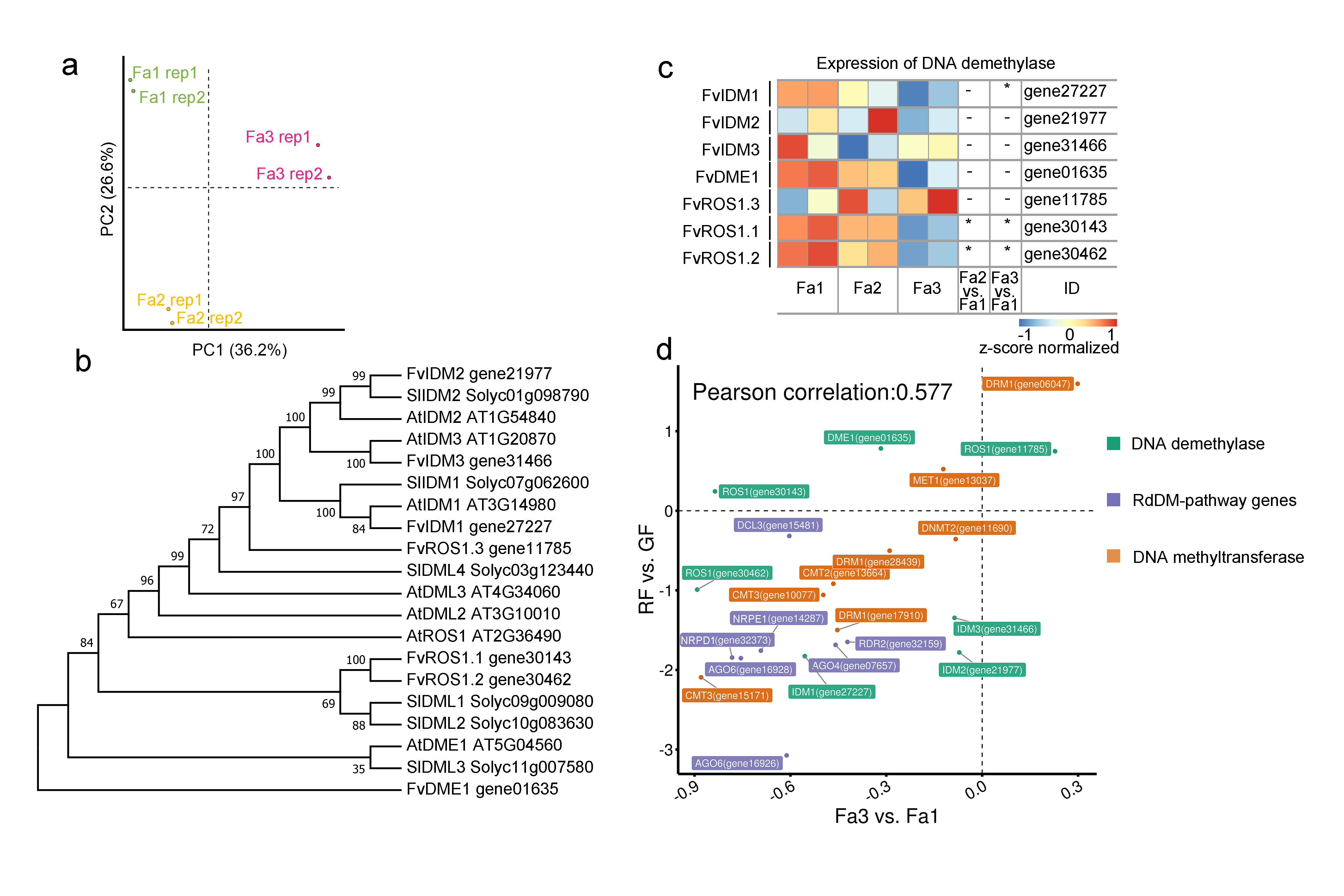


# Figure S4. Expression of genes involved in DNA demethylation.

1. PCA showing consistency between biological replicates of fruits at each stage. Transcriptomes of Fa1~Fa3 are shown.
2. Phylogenetic analysis of genes involved in DNA demethylation pathway in strawberry (Fv), tomato (Sl) and *Arabidopsis* (At).
3. Heatmap showing transcript levels of genes involved in DNA demethylation pathway in Fa1~Fa3 (* adjusted *P* value < 0.05, as determined using the DESeq).
4. High consistency (Pearson correlation coefficient = 0.58) of the expression level of genes involved in DNA methylation and demethylation during ripening determined by transcriptomic data in this study and determined using public data. Transcriptomic data from stages Fa1 and Fa3 in this study and red fruit (RF) and green fruit (GF) in published study (see methods for further description) were used.


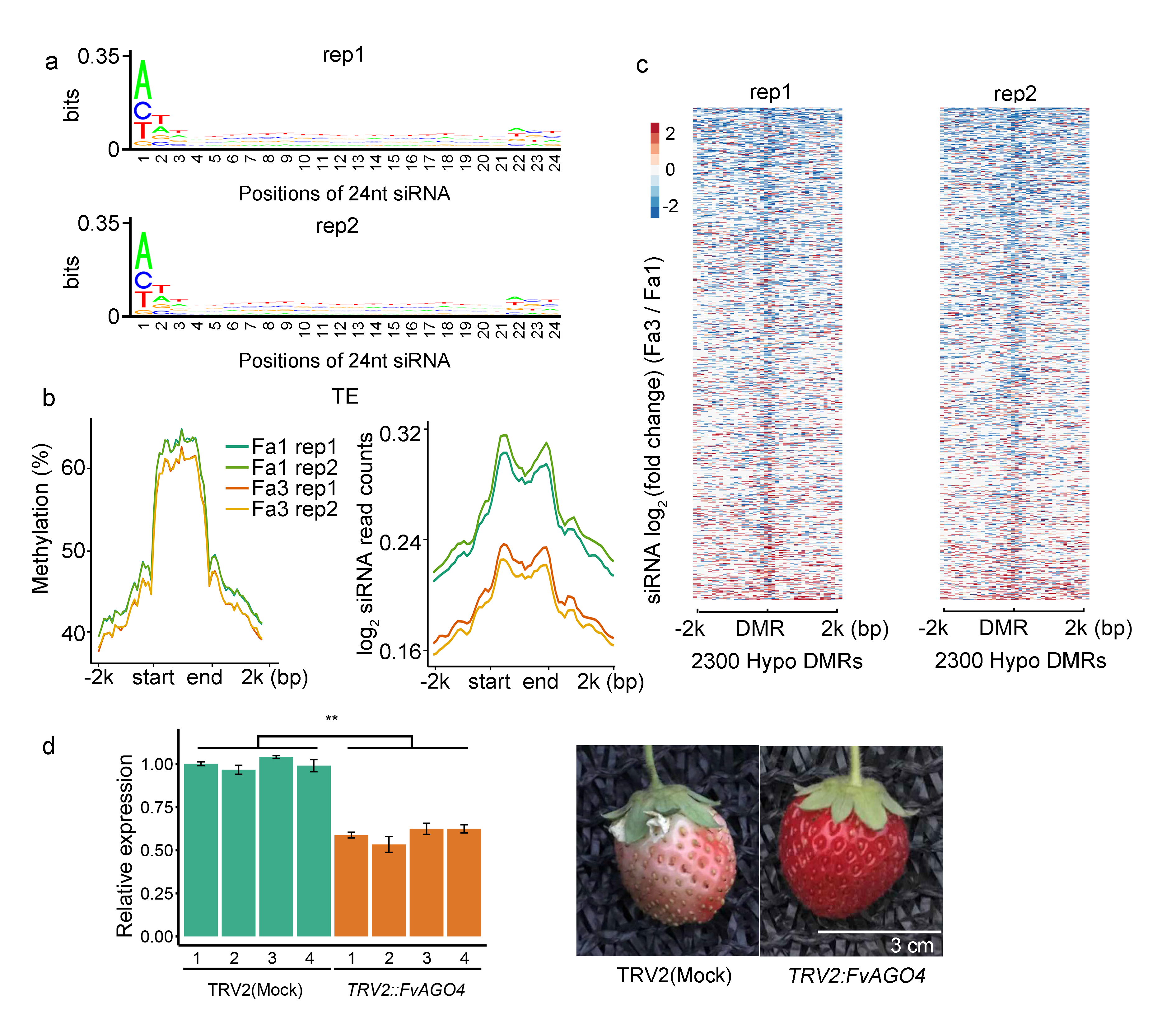


# Figure S5. Analyses of siRNAs in strawberry fruits.

1. Relative nucleotide bias at each position of 24-nt siRNA. Two biological replicates were used.
2. Profiles of DNA methylation (left panel) and siRNA (right panel) surrounding TEs in Fa1 and Fa3. Shown are two biological replicates from stages Fa1 and Fa3.
3. Heatmap showing changes of siRNA level surrounding each hypo-DMR. Two biological replicates were used.
4. TRV2-induced gene silencing of *FvAGO4* in strawberry fruits. RT-qPCR analysis of *FvAGO4* in TRV2 control and TRV2:*FvAGO4* fruits are shown on the left. Pictures of TRV2 control and TRV2:*FvAGO4* fruits are shown on the right.


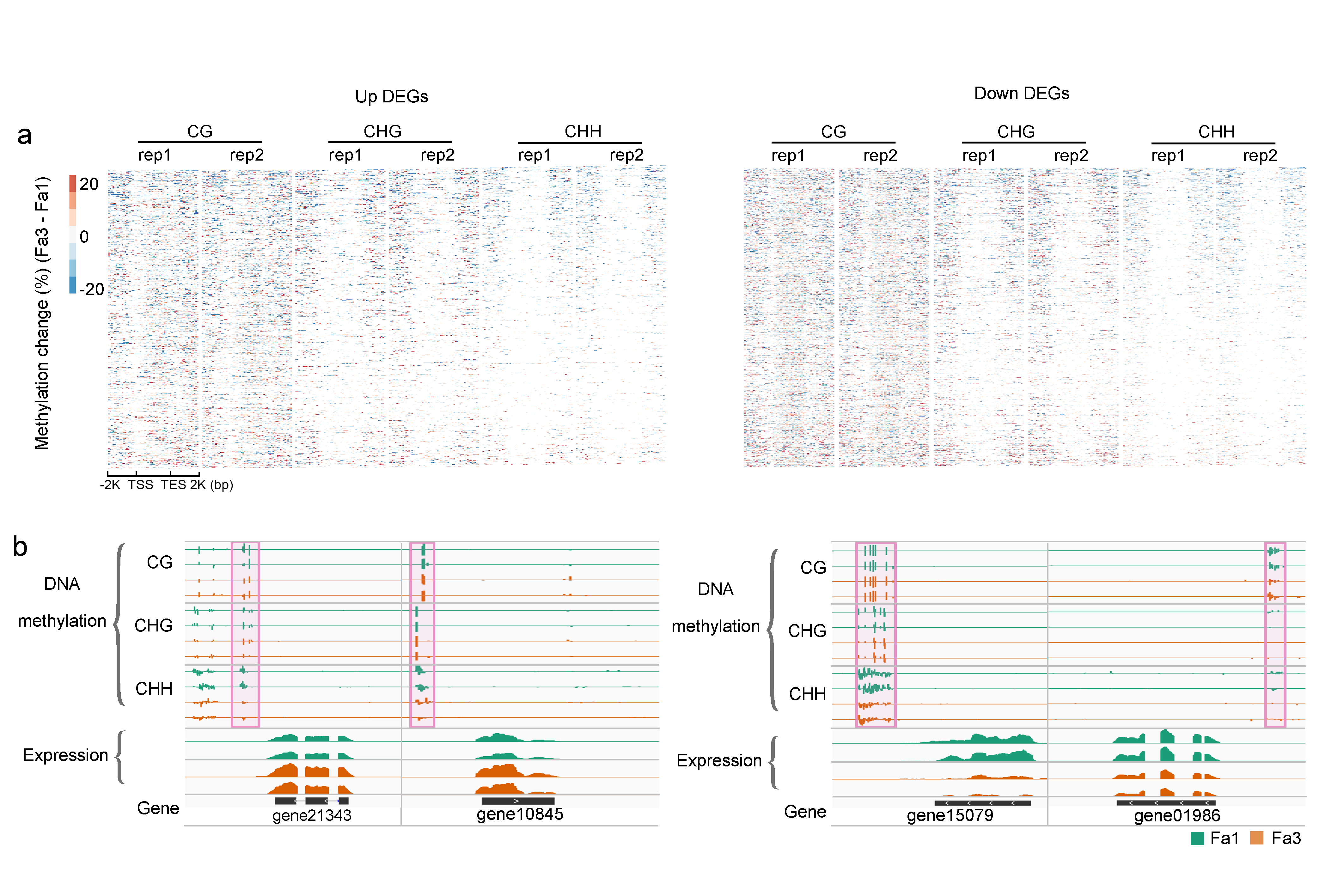


# Figure S6. Association between gene expression and DNA methylation during ripening.

1. Heatmap showing DNA methylation changes of ripening-related genes in mCG, mCHG, and mCHH context. DNA methylation changes (Fa3-Fa1) of 1,417 down DEGs (left panel) and 899 up DEGs (right panel) are shown. Two biological replicates were used.
2. IGB display of DNA methylation levels (CG, CHG and CHH context) and transcript levels of two up DEGs (upper panel) and two down DEGs (lower panel) in Fa1 and Fa3. Hypo-DMRs are boxed. Two biological replicates were used.


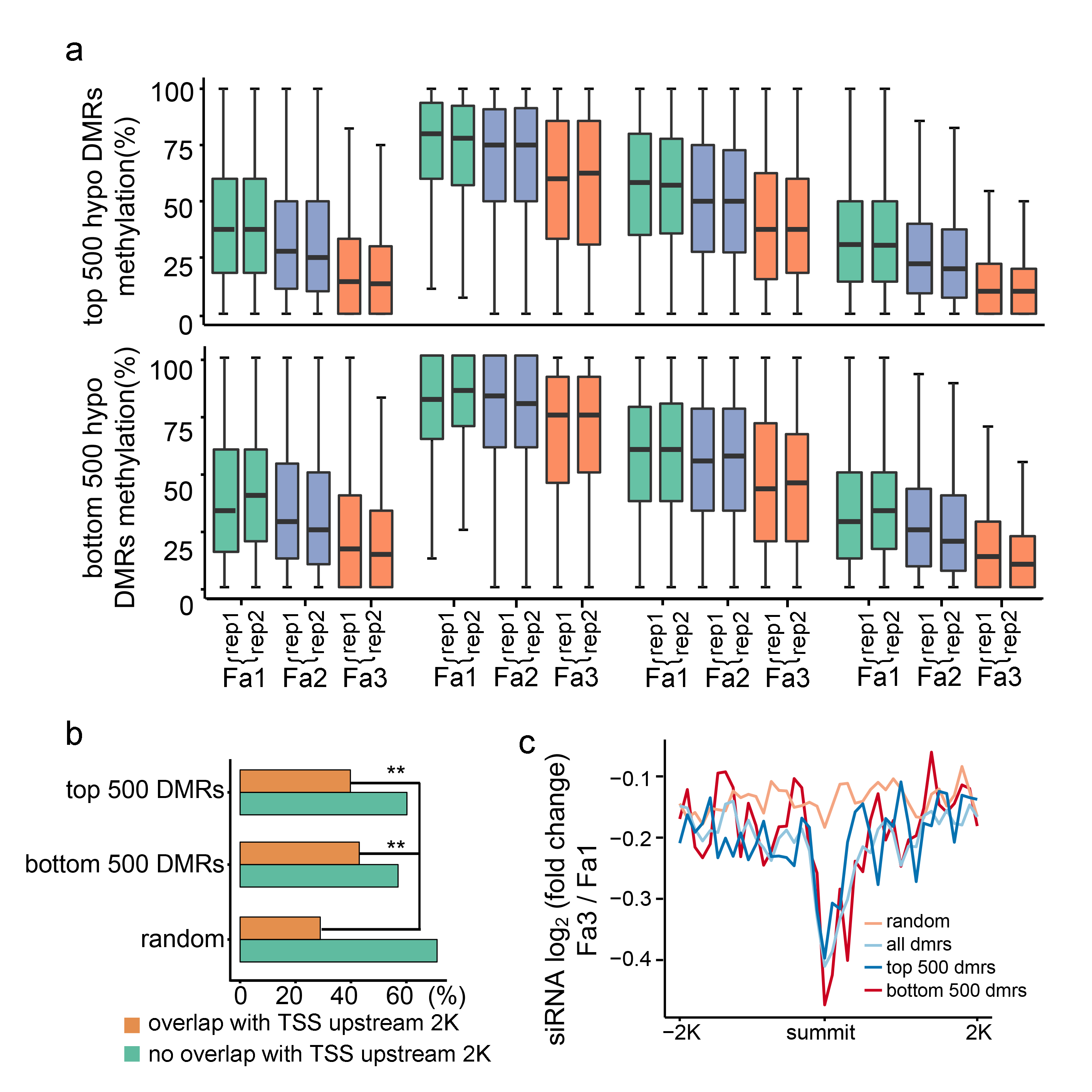


# Figure S7. Repeatability between replicates using robust index.

1. Box plots showing that both top 500 and least 500 credible hypo DMRs show a gradual decrease in DNA methylation during fruit ripening.
2. Both top 500 and least 500 credible hypo DMRs are enriched in promoter regions. Random, randomly selected regions.
3. Both top 500 and least 500 credible hypo DMRs show decreased siRNA accumulation during ripening.
