# Supplementary material for: Downregulation of RdDM during strawberry fruit ripening
Source: Genome Biol. 2018 Dec 4;19:212. doi: 10.1186/s13059-018-1587-x (PMC6280534; doi:10.1186/s13059-018-1587-x)
Supplement: Supplementary file 9 — Review history. (DOCX 57 kb) [file 13059_2018_1587_MOESM9_ESM.docx]

Review History

**First round of review**

**Reviewer 1**

**Are the methods appropriate to the aims of the study, are they well described, and are necessary controls included?**
Replicates should be treated as replicates throughout the study and not combined.

**Are the conclusions adequately supported by the data shown?**

No, There is no direct link between RdDM and ripening presented in this study although it is stated numerous times throughout the manuscript that they're linked.

**Are sufficient details provided to allow replication and comparison with related analyses that may have been performed?**

Yes

**Does the work represent a significant advance over previously published studies? Please explain.**

No

**Is the paper of broad interest to others in the field, or of outstanding interest to a broad audience of biologists?**

No.

Comments to authors:

This paper describes an epigenome analysis throughout ripening in strawberry. Single base resolution methylomes and small RNA seq were used and revealed that there is a significant amount of hypomethylation during ripening. This hypomethylation was associated with a decrease in 24nt siRNAs. Interestingly numerous components of the RdDM pathway were transcriptionally down regulated during this developmental progression. These results led the authors to conclude that RdDM regulates the ripening process in strawberry.

Although the observations of hypomethylation, decreased siRNAs and decreased expression of RdDM pathway components throughout the ripening process is interesting, it does not provide evidence that RdDM regulates ripening. There is no causal data provided in this study to support a direct link between RdDM and ripening. In general, there is a lot of overfitting of the data based on pioneering work in tomato ripening. The loss of methylation/siRNAs could be mutually exclusive from the ripening process.

Lastly, although there is clear decreased in siRNAs/methylation there is still siRNAs and methylation at these loci. What is the significance of these identified differences? Identifying mutations in genes in an RdDM component and finding an altered ripening time is required. I realize this is not a trivial experiment, but without it the assertions of the paper are not supported. This leaves the reader with an interesting decrease in siRNAs/methylation that happens to occur during ripening. It doesn't mean they are causally linked.

Further comments are provided below:

How many CHH methylated regions are there in the genome that are likely targets of RdDM meaning they have 24nt siRNAs, they are not in bodies of long TEs or in the pericentromeric regions (as these would likely be methylate by CMT2)? If RdDM components are downregulated it seems there would be much more than 2300 hypo DMRs. What proportion of mCHH regions lose methylation and why do other mCHH regions in euchromatin not lose it.

Same question for CHG. If CMT3 is down-regulated why is there not a stronger decrease in CHG genome-wide?

Line 300. Why is there no statistical test for a positive correlation presented? It seems the DEGs are found next to hypo DMRs, but there is no preference for up or down regulation. This would suggest the change in methylation is not causal for the differences in gene expression. Perform the same analysis for hyper-DMRs. How many genes are Up versus down? What is the conclusion about these loci?

Line 308. Perform the GO analysis with DEGs (Up or down) regardless of their methylation status. Same with hyper DMR associated with up or down. There are many permutations that can be tested, but the focus is always on the class that the authors want to be involved in this process. There needs to be a more unbiased analysis of these data.

Line 178. Even though replicates were produced they were not used as replicates in any of the analyses. Therefore, there is no replication of the data in this study. If the authors require more statistical power they should sequence their individual replicates to greater depth.

Line 192 - there is no statistical support to show that hypo DMRs were enriched. Even if they are this is not surprising as most methylation CHH in euchromatin is found near genes. What does "Genome" represent in Figure 3A? Is this the distribution of methylated regions throughout the genome?

Can the authors explain why the "random" pattern in 3C is not random? Why would there be an enrichment in gene bodies?

Line 185. The data for hyper DMRs should also be presented (even if just as a supplementary figure).

Line 400 - There is no data presented in this manuscript to support this statement. It is an association at this point.

Lines 140-155 are unnecessary as the information provided are confirmatory and expected. This information is not relevant to the title of the study. Figure 1 is unnecessary and could be moved to the supplement.

The title of the paper is inaccurate as the data provided do not determine that RdDM is causally related to strawberry fruit ripening.

All of the sequencing data should be deposited to a public data repository.

A supplementary file should be provided that includes sequencing summary data for siRNA and methylome sequencing. This should include, raw read number and aligned read number, types of reads, alignment percentage and for methylomes it should show the bisulfite conversion efficiency.

**Reviewer 2**

**Are the methods appropriate to the aims of the study, are they well described, and are necessary controls included?**

No.

There is no details about how the library was constructed, or the details of their mapping strategies.

There is also no GEO submission to display their data.

**Are the conclusions adequately supported by the data shown?**

Mostly yes.

I would also add that RdDM is not 100% necessary for the loss of DNA methylation in this paper, so there must be other mechanisms at play. In fact, RdDM may be downstream of the real mechanism starting this loss of methylation, and these alternatives should be explored (including in the title).

**Are sufficient details provided to allow replication and comparison with related analyses that may have been performed?**

NO.

1. There is no description of how the libraries were generate.

2. There are no sequencing statistics, such as conversion rates for bisulfitetreated

libraries.

3. There is no an adequate description of mapping

4. the data has not been made available to the reader.

**Does the work represent a significant advance over previously published studies? Please explain.**

Yes. This analysis in this species has never been performed.

**Is the paper of broad interest to others in the field, or of outstanding interest to a broad audience of biologists?**

Yes: There seems to be conservation of decreased methylation during ripening of different fruits (although the mechanisms seem different)

**Comments to author**

This is a review of the manuscript entitled "RdDM is Involved in epigenetic regulation of strawberry fruit ripening" submitted to Genome Biology by Cheng et al. In this manuscript the authors observe the process of strawberry ripening, and find a general reduction of DNA methylation and small RNAs. They also observe a change in gene expression during ripening that they associate with the small RNA / DNA methylation change. Overall, I believe this manuscript to be interesting, by lacking many necessary controls. In addition, the data availability and openness to how the libraries where generated and handled are grossly sub-standard. I suggest 13 changes that need to be completed.

Major changes to be made

1.	I do not agree with line 177-178 that the statistical power is greater if biological replicates are pooled and treated as one. This may be true for calling DMRs, but for demonstrating that a change at a DMR is biologically reproducible, both biological replicates must be shown for Figures: 3 (the entire Figure), S3 (I see no correlation), S5A (the 5' end enrichment is very weak), S5C (the trend is very week), S6A - (the trend is very weak). For each of these, the observed pattern is so weak that I need to see the data for more than one biological replicate to be convinced.

2.	The authors explore expression, small RNAs and DNA methylation. However, there is a lack of cross-over analysis between these data types. This must be expanded. For example: investigate the small RNAs at the gene flanks that lose CHH methylation, and investigate the CHH methylation at loci that have reduced small RNAs in Fa3 fruits compared to loci that do not change.

3.	I do not agree with line 360, where the authors explain that de-methylation becomes dominant over methylation by RdDM. Instead, there is no evidence that de-methylation is involved at all, and rather just DNA methylation is reduced. The fact that this lack of methylation must be due to RdDM AND non-RdDM mechanisms is not emphasized enough, including in the title. Even in an RdDM null mutant, the fruit would still undergo decreased methylation due to the other mechanisms affecting CHG (likely through CMT3). Model of passive DNA de-methylation without the positive feedback cycle of CMT3 / histone modification activity after DNA replication need to be mentioned / emphasized.

4.	There is not nearly enough detail in the Methods section regarding deep sequencing. For example, library preparation is barely mentioned as "…library construction using Illumina's standard methylation analysis protocol…" or "…the libraries were constructed and sequenced at the…" or "…libraries were constructed and sequenced at the...". There are hundreds of these protocols, and the reader needs to know the exact details. For the methylation libraries, what is the conversion rate of the bisulfite? There are many details that need to be added that are critical factors that must be added. For mapping, the authors write "…Uniquely and properly mapped paired-end reads…". This is a very non-scientific sentence. How were multi-mapping sequences handled? The authors must provide the details of how the mapping program was run so it can be repeated in the future.

5.	For Figure 6C, please separate the DNA methylation analysis by cytosine context: CG, CHG, CHH.

6.	Figure 3A needs to have a statistical analysis of observed compared to expected. There needs to be a proper statistical / probability analysis of enrichment.

7.	I am not convinced of the stated result by Figure 5B. I do not see a trend. Perhaps a moving-average trend line would show this?

8.	Figure 2A,B,C does not make sense and needs to be redone correctly. The authors define hypo DMRs, and then show that these regions lose DNA methylation. Of course they do, and they do by definition as they are "hypo DMRs", because they were the pre-selected regions of the genome. This is circular reasoning.

9.	How, in Figure 3A, can only ~10% of the genome be annotated as TEs, while 28% of the genome is annotated as a transcriptional start site? Something in this figure is very wrong. One quarter of the annotated genome should not be gene start sites.

10.	For Figure 3C, please focus on the individual cytosine contexts, CG, CHG, and CHH, and please describe what the randomly selected grey line represents. There needs to be a full description of what was informatically done to create a "randomly selected" pattern that is so striking.

11.	In Figure 6B, only about 1/3 of the loci examined have the expected pattern. This very easily could be random. The authors must compare genes are not up nor down DEGs and see if randomly 1/3 of those also have decreased methylation, as this would prove that the changes in methylation are randomly associated with gene expression. These types of controls should be used throughout the manuscript.

12.	The same comment as #11 but for figure S6A. It looks to me that only 1/3 of the loci show the affect, which could be random. The authors need to ask what percent show the expected trend of DNA loss compared to regions with no change and with increased methylation? It looks to me that 1/3 is random.

13.	I did not find where the data was publically available. A GEO submission must accompany this publication.

**Authors Response**

**Point-by-point responses to the reviewers’ comments:**

Point-by-point response to reviewers

Reviewer reports:

Reviewer #1: This paper describes an epigenome analysis throughout ripening in strawberry. Single base resolution methylomes and small RNA seq were used and revealed that there is a significant amount of hypomethylation during ripening. This hypomethylation was associated with a decrease in 24nt siRNAs. Interestingly numerous components of the RdDM pathway were transcriptionally down regulated during this developmental progression. These results led the authors to conclude that RdDM regulates the ripening process in strawberry.

Although the observations of hypomethylation, decreased siRNAs and decreased expression of RdDM pathway components throughout the ripening process is interesting, it does not provide evidence that RdDM regulates ripening. There is no causal data provided in this study to support a direct link between RdDM and ripening. In general, there is a lot of overfitting of the data based on pioneering work in tomato ripening. The loss of methylation/siRNAs could be mutually exclusive from the ripening process.

Lastly, although there is clear decreased in siRNAs/methylation there is still siRNAs and methylation at these loci. What is the significance of these identified differences? Identifying mutations in genes in an RdDM component and finding an altered ripening time is required. I realize this is not a trivial experiment, but without it the assertions of the paper are not supported. This leaves the reader with an interesting decrease in siRNAs/methylation that happens to occur during ripening. It doesn't mean they are causally linked.

*Answer: We appreciate the reviewer’s criticism. Indeed, our original manuscript did not provide evidence for a causal relationship between DNA methylation/siRNA loss and fruit ripening. To address this question, we performed new experiments as shown in figures 3A, 3B and S5. We treated young strawberry fruits with a DNA methylation inhibitor (5-Azacytidine), and found that the treatment caused reduced DNA methylation levels at tested genes and an early ripening phenotype in strawberry fruits (Fig. 3A and 3B). The data suggest that a loss of DNA methylation can induce strawberry fruit ripening, and thus indicate a causal link between DNA methylation loss and ripening. In addition, we used TRV-mediated gene silencing to down-regulate FvAGO4, an important RdDM factor that is abundantly expressed in immature strawberry fruits, and observed an early ripening phenotype in FvAGO4 silenced fruits (Fig. S5), supporting that a reduced RdDM activity is involved in strawberry ripening.*

Further comments are provided below:

How many CHH methylated regions are there in the genome that are likely targets of RdDM meaning they have 24nt siRNAs, they are not in bodies of long TEs or in the pericentromeric regions (as these would likely be methylate by CMT2)?

*Answer: We identified 370,630 24nt siRNA clusters in the strawberry genome, and 345,828 of them are not located in the bodies of long TEs. We added the following description in the main text:*

*“The biogenesis of 24-nt siRNA depends on RdDM. However, in Arabidopsis, it is known that long TEs usually have siRNAs in their body regions, even though their body methylation relies on CMT2 [3]. In strawberry, we identified 370,630 24-nt siRNA clusters (Table S4); 345,828 of these clusters are not located in long TE bodies, and thus can be considered as canonical RdDM target regions.”*

If RdDM components are downregulated it seems there would be much more than 2300 hypo DMRs. What proportion of mCHH regions lose methylation and why do other mCHH regions in euchromatin not lose it. Same question for CHG. If CMT3 is down-regulated why is there not a stronger decrease in CHG genome-wide?

*Answer: The loss of existing DNA methylation can be caused by passive DNA demethylation (which requires cell division and DNA replication) and/or active DNA demethylation (which requires the ROS1 family demethylases). There is little cell division during fruit ripening, thus any loss of DNA methylation would require active DNA demethylation because without it, the DNA methylation already there cannot disappear even when there is no new methylation. Although the DNA demethylase genes are not up-regulated during strawberry fruit ripening, the overall loss of DNA methylation indicates that they are functional during the ripening process. It is known that DNA demethylases erase DNA methylation at some but not all genomic regions targeted by RdDM and/or CMT3 (Tang et al., 2016 Nature Plants). Therefore, the genomic pattern of DNA methylation loss is determined by not only reductions in methylation pathways such as RdDM and CTM3 but also where the DNA demethylases are targeted. This point is now elaborated in the revised manuscript (page15, first paragraph).*

Line 300. Why is there no statistical test for a positive correlation presented?

*Answer: The “positive correlation” in line 300 referred to an observation that was reported in a previous tomato study using DNA demethylase mutants (Lang et al 2017 PNAS). Since we do not have strawberry DNA demethylase mutants, we have changed the description in the revised manuscript as follows:*

*“However, for down-regulated DEGs, the DNA hypomethylation corresponded to decreased expression during ripening (Fig. 6b, 6c). A recent study using DNA demethylase mutants revealed that DNA demethylation is associated with the inactive expression for several hundred genes during tomato ripening [14]. How DNA demethylation causes down regulation of gene expression is still unclear in both tomato and strawberry.”*

It seems the DEGs are found next to hypo DMRs, but there is no preference for up or down regulation. This would suggest the change in methylation is not causal for the differences in gene expression. Perform the same analysis for hyper-DMRs. How many genes are Up versus down? What is the conclusion about these loci?

*Answer: We performed the same analysis for hypermethylated genes, and found 67 up-DEG and 110 down-DEGs. So for both hypo- and hyper-DMR associated DEGs, there is no preference for up or down regulation. Similarly, in tomato fruits, hypo DMR-associated DEGs do not have a preference for down or up regulation (Lang et al 2017 PNAS). However, the tomato study found using DNA demethylase mutants that the hypomethylation is required for both the up and down regulation of gene expression (Lang et al 2017 PNAS). In our revised manuscript, we examined the methylation and expression levels of two up-regulated DEGs (gene10875 and gene30616) in 5-Aza treated samples. Compared with mock treatment, the promoter regions of these two genes were hypomethylated and their expression was upregulated in 5-Aza treated fruits, suggesting a repressive role of DNA methylation for these two genes (Figure 3B).*

Line 308. Perform the GO analysis with DEGs (Up or down) regardless of their methylation status. Same with hyper DMR associated with up or down. There are many permutations that can be tested, but the focus is always on the class that the authors want to be involved in this process. There needs to be a more unbiased analysis of these data.

*Answer: We thank the reviewer for this suggestion. We performed GO analysis with up and down DEGs regardless of their methylation status. The analysis showed that ripening-related GO terms were enriched in the hypomethylated but not non-hypomethylated DEGs (Figure 6D and Table S6), supporting a role of DNA hypomethylation in the ripening process.*

Line 178. Even though replicates were produced they were not used as replicates in any of the analyses. Therefore, there is no replication of the data in this study. If the authors require more statistical power they should sequence their individual replicates to greater depth.

*Answer: In the revised manuscript, we displayed data of two biological replicates in Figures 1d, 2a, 2b, 5d and S6.*

Line 192 - there is no statistical support to show that hypo DMRs were enriched. Even if they are this is not surprising as most methylation CHH in euchromatin is found near genes. What does "Genome" represent in Figure 3A? Is this the distribution of methylated regions throughout the genome?

*Answer: In the revised manuscript, we preformed statistical test and found that hypo-DMRs are enriched at the 2 kb region upstream of TSS (Fig. 3d). "Genome" represents the distribution of all genomic sequences in the whole genome.*

Can the authors explain why the "random" pattern in 3C is not random? Why would there be an enrichment in gene bodies?

*Answer: Thank you for pointing this out. There was bias in our previous analysis, i.e. the bin-size is different between gene body and surrounding regions (due to different gene length) (left panel below). In the revised manuscript, we resolved the issue by plotting instead DMR distribution around TSS and TES separately (revised Fig. 3F).*

Line 185. The data for hyper DMRs should also be presented (even if just as a supplementary figure).

*Answer: In the revised MS, we presented data for hyper DMRs in figure S2D.*

Line 400 - There is no data presented in this manuscript to support this statement. It is an association at this point.

*Answer: In the revised MS, we have included data from DNA methylation inhibitor treatment (Fig.3A, 3B) to support the statement that DNA hypomethylation regulates the ripening of the non-climacteric fruit. Also, we used TRV-mediated gene silencing to down-regulate FvAGO4, an important factor in RdDM, and observed an early ripening phenotype in FvAGO4- silenced fruits, suggesting that the down-regulation of RdDM is involved in strawberry ripening (Fig. S5).*

Lines 140-155 are unnecessary as the information provided are confirmatory and expected. This information is not relevant to the title of the study. Figure 1 is unnecessary and could be moved to the supplement.

*Answer: We have moved Figure 1 to the supplement. The DNA methylome of strawberry fruit has never been characterized before, so some description of the general features of the DNA methylome is kept in the revised MS.*

The title of the paper is inaccurate as the data provided do not determine that RdDM is causally related to strawberry fruit ripening.

*Answer: In the revised MS, we used TRV-mediated gene silencing to downregulate FvAGO4, an important factor in RdDM, and observed an early ripening phenotype in FvAGO4-silenced fruits, suggesting that the downregulation of RdDM is involved in strawberry ripening (Fig. S5). We agree that more data are needed to establish a causal relationship between RdDM and strawberry fruit ripening, so we have changed the title to “Down-regulation of RdDM during strawberry fruit ripening”.*

All of the sequencing data should be deposited to a public data repository.

*Answer: We have deposited all sequencing data to NCBI, and the data accession numbers are added in the revised manuscript.*

A supplementary file should be provided that includes sequencing summary data for siRNA and methylome sequencing. This should include, raw read number and aligned read number, types of reads, alignment percentage and for methylomes it should show the bisulfite conversion efficiency.

*Answer: In the revised MS, we have added the suggested sequencing summary data in the main text and supplementary files (Table S1).*

Reviewer #2: This is a review of the manuscript entitled "RdDM is Involved in epigenetic regulation of strawberry fruit ripening" submitted to Genome Biology by Cheng et al. In this manuscript the authors observe the process of strawberry ripening, and find a general reduction of DNA methylation and small RNAs. They also observe a change in gene expression during ripening that they associate with the small RNA / DNA methylation change. Overall, I believe this manuscript to be interesting, by lacking many necessary controls.

In addition, the data availability and openness to how the libraries where generated and handled are grossly sub-standard. I suggest 13 changes that need to be completed.

Major changes to be made

1. I do not agree with line 177-178 that the statistical power is greater if biological replicates are pooled and treated as one. This may be true for calling DMRs, but for demonstrating that a change at a DMR is biologically reproducible, both biological replicates must be shown for Figures: 3 (the entire Figure), S3 (I see no correlation), S5A (the 5' end enrichment is very weak), S5C (the trend is very week), S6A - (the trend is very weak). For each of these, the observed pattern is so weak that I need to see the data for more than one biological replicate to be convinced.

*Answer: According to the reviewer’s suggestion, in the revised manuscript, we displayed data of two biological replicates in revised figures 2 (corresponding to original figure 3),S5A, S5C and S6A, and the two biological replicates showed consistent trends. Figure S3 shows hyper DMR associated genes as negative control of hypo DMR associated genes, so we expected to see “no correlation”.*

2. The authors explore expression, small RNAs and DNA methylation. However, there is a lack between these data types. This must be expanded. For example: investigate the small RNAs at the gene flanks that lose CHH methylation, and investigate the CHH methylation at loci that have reduced small RNAs in Fa3 fruits compared to loci that do not change.

*Answer: In the revised manuscript, we added the suggested analysis, which showed a decreased CHH methylation level at genomic regions with reduced siRNA levels, and decreased siRNAs at the hypo DMRs (Figure 5e).*

3. I do not agree with line 360, where the authors explain that demethylation becomes dominant over methylation by RdDM. Instead, there is no evidence that de-methylation is involved at all, and rather just DNA methylation is reduced. The fact that this lack of methylation must be due to RdDM AND non-RdDM mechanisms is not emphasized enough, including in the title. Even in an RdDM null mutant, the fruit would still undergo decreased methylation due to the other mechanisms affecting CHG (likely through CMT3). Model of passive DNA de-methylation without the positive feedback cycle of CMT3 / histone modification activity after DNA replication need to be mentioned / emphasized.

*Answer: We added the following discussion of the possibility that non-RdDM mechanism could also contribute to DNA hypomethylation during strawberry fruit ripening: “In addition, strawberry CMT3 was down-regulated during strawberry, which could also contribute to DNA hypomethylation.”*

*The loss of existing DNA methylation can be caused by passive DNA demethylation (which requires cell division and DNA replication) and/or active DNA demethylation (which requires the ROS1 family demethylases). There is little cell division during fruit ripening, thus any loss of DNA methylation would require active DNA demethylation because without it the DNA methylation already there cannot disappear even when there is no new methylation. Although the DNA demethylase genes are not up-regulated during strawberry fruit ripening, the overall loss of DNA methylation indicates that they are functional during the ripening process. It is known that DNA demethylases erase DNA methylation at some but not all genomic regions targeted by RdDM and/or CMT3 (Tang et al., 2016 Nature Plants). Therefore, the genomic pattern of DNA methylation loss is determined by not only reductions in methylation pathways such as RdDM and CTM3 but also where the DNA demethylases are targeted. This point is now elaborated in the revised manuscript (page15, first paragraph).*

4. There is not nearly enough detail in the Methods section regarding deep sequencing. For example, library preparation is barely mentioned as "…library construction using Illumina's standard methylation analysis protocol…" or "…the libraries were constructed and sequenced at the…" or "…libraries were constructed and sequenced at the...". There are hundreds of these protocols, and the reader needs to know the exact details. For the methylation libraries, what is the conversion rate of the bisulfite? There are many details that need to be added that are critical factors that must be added. For mapping, the authors write "…Uniquely and properly mapped paired-end reads…". This is a very non-scientific sentence. How were multi-mapping sequences handled? The authors must provide the details of how the mapping program was run so it can be repeated in the future.

*Answer: We added detailed information about sequencing and mapping program in the method of revised manuscript. We also included conversion rates information in the main text of revised manuscript. “The libraries of whole genome bisulfite sequencing were prepared using NEBNext Ultra II DNA Library Prep Kit for Illumina (New England Biolabs) and Epitect Plus DNA Bisulfite Kit (Qiagen). Briefly, 1 μg of genomic DNA was sonicated into 200~500 bp fragments on a Covaris M220. The fragmented DNA was subjected to end repair, A-tailing, and adaptor ligation following the manufacturer’s instructions of the NEBNext Ultra II DNA Library Prep Kit. The adapter ligated products were then treated with sodium bisulfite on a thermocycler using Epitect Plus DNA Bisulfite Kit (Qiagen) with the following program: 95ºC 5 min, 60ºC 25 min, 95ºC 5 min, 60ºC 85 min, 95ºC 5 min, 60ºC 175 min, 3x (95ºC 5 min, 60ºC 180 min), hold at 20ºC. The BS-treated DNA was then cleaned up using Epitect Plus DNA Bisulfite Kit (Qiagen) and PCR-amplified using the KAPA HiFi Hotstart ReadyMix for 6 cycles. The amplified libraries were finally cleaned up using magnetic beads from Vazyme. The libraries were sequenced on a HiSeq2500 (Illumina) in paired end 125 bp mode using the HiSeq PE Cluster Kit v4 (Illumina) and HiSeq SBS Kit v4 (250 cycles) (Illumina) following the manufacturer’s instructions at the Genomics Core Facility of the Shanghai Center for Plant Stress Biology, Chinese Academy of Sciences.”*

5. For Figure 6C, please separate the DNA methylation analysis by cytosine context: CG, CHG, CHH.

*Answer: According to reviewer’s suggestion, we separated the DNA methylation analysis by contexts in the revised MS (Figure S6).*

6. Figure 3A needs to have a statistical analysis of observed compared to expected. There needs to be a proper statistical / probability analysis of enrichment.

*Answer: According to reviewer’s suggestion, we performed Fisher’s exact test in the revised manuscript, and found that hypo-DMRs are enriched at the upstream 2 kb regions of TSS (Fig. 3d).*

7. I am not convinced of the stated result by Figure 5B. I do not see a trend. Perhaps a moving-average trend line would show this?

*Answer: We added a moving-average trend line in revised figure 5B, and it showed that strawberry 24-nt siRNAs are preferentially located in promoter and 3’ regulatory regions around genes, but are more widely spread along TE bodies and flanking regions.*

8. Figure 2A,B,C does not make sense and needs to be redone correctly. The authors define hypo DMRs, and then show that these regions lose DNA methylation. Of course they do, and they do by definition as they are "hypo DMRs", because they were the pre-selected regions of the genome. This is circular reasoning.

*Answer: We apologize for the confusion. We generated figure 2 to address several points:*

*1) To show that DNA methylation was decreased consistently in TWO biological replicates;*

*2) To examine whether the loss of DNA methylation has any context preference. Through figures 2A and 2C, we found that the decreased DNA methylation occurred in all three contexts;*

*3) For figure 2C, we plotted all cytosines in all DMRs, but not only in hypo DMRs, so the left skewed peak helped to support the statement that hypomethylation is more prevalent than hypermethylation during ripening.*

*4) For figure 2B, we wanted to show the change of DNA methylation and siRNAs at several representative regions.*

*In the new MS, we clarified these points in the main text as following: “To investigate whether the hypomethylation preferentially occurred in any sequence context, we examined DNA methylation levels of hypo-DMRs in two biological replicates in CG, CHG and CHH contexts. As shown in Figure 2a, the two biological replicates showed the same trend of change that DNA methylation was gradually decreased in all three contexts from Fa1 to Fa3 (Fig. 2a). The methylation levels of several representative hypo-DMRs are shown in Figures 2b and S2c. We calculated the DNA methylation levels for each cytosine within all DMRs, which showed a left skewed pattern, suggesting that most cytosines were hypomethylated in Fa3 compared to Fa1 in all three contexts (Fig. 2c).”*

9. How, in Figure 3A, can only ~10% of the genome be annotated as TEs, while 28% of the genome is annotated as a transcriptional start site? Something in this figure is very wrong. One quarter of the annotated genome should not be gene start sites.

*Answer: Sorry for the confusion. It should be promoter (TSS upstream 2kb) instead of TSS, which we corrected in the revised manuscript.*

10. For Figure 3C, please focus on the individual cytosine contexts, CG, CHG, and CHH, and please describe what the randomly selected grey line represents.

*Answer: Sorry for the confusion. We plotted the DMR density but not DNA methylation level along genes in figure 3C, so we were not able to focus on individual cytosine contexts.*

There needs to be a full description of what was informatically done to create a "randomly selected" pattern that is so striking.

*Answer: Thank you for pointing this out. There was bias in our previous analysis, i.e. the bin-size is different between gene body and surrounding regions (due to different gene length) (left panel below). In the revised manuscript, we resolved the issue by plotting instead DMR distribution around TSS and TES separately (revised Fig. 3F).*

11. In Figure 6B, only about 1/3 of the loci examined have the expected pattern. This very easily could be random. The authors must compare genes are not up nor down DEGs and see if randomly 1/3 of those also have decreased methylation, as this would prove that the changes in methylation are randomly associated with gene expression. These types of controls should be used throughout the manuscript.

12. The same comment as #11 but for figure S6A. It looks to me that only 1/3 of the loci show the affect, which could be random. The authors need to ask what percent show the expected trend of DNA loss compared to regions with no change and with increased methylation? It looks to me that 1/3 is random.

*Answer: We agree with the reviewer that the DNA methylation change is also associated with many non-DEGs. In fact, a similar pattern was also observed for tomato fruit ripening (~28,000 DMRs during tomato ripening are located at promoters of ~6,000 genes, but only 1,200 of the 6,000 are DEGs) (Lang et al PNAS 2017). In both tomato and strawberry fruits, DNA methylation changes at DEGs as well as many non-DEGs during ripening, thus the effect on gene expression seems to be random. However, data from tomato sldml2 mutants and DNA methylation inhibitor treatment data from both plants support that the change of DNA methylation is important for proper ripening (Lang et al PNAS 2017 and figure3). Our GO term analysis revealed that ripening-related terms are enriched in hypo-methylated DEGs but not in non-hypomethylated DEGs (Figure 6d). Our previous work using DNA demethylase mutants in tomato (Lang et al PNAS 2017) showed that DNA hypomethylation can cause gene repression as well as activation. For the many genes where DNA hypomethylation does not cause changes in expression, it is likely that the methylation changes are not sufficient to cause expression changes in the fruits and under the experimental conditions; it is possible that for some of these genes, the methylation changes can cause expression changes in other tissues and/or under other biotic and abiotic conditions.*

13. I did not find where the data was publically available. A GEO submission must accompany this publication.

*Answer: We have deposited all sequencing data to NCBI, and the data accession numbers have been added in the new MS.*

**Second round of review**

**Reviewer 1**

The addition of functional data to support the conclusions of this manuscript significantly improve the quality of the study. My only remaining concern is that the authors are still not incorporating replicate data into their genome-wide analyses. Showing replicate data is very different from incorporating it into analyses.

**Authors Response**

**Point-by-point responses to the reviewers’ comments:**

We appreciate the reviewer’s comments. In our further revised manuscript, we added new analysis to incorporate the biological replicates into genome-wide analysis. We calculated a robust index using the following formula for each DMR to measure the repeatability between the replicates.

Robust index = (|〖log〗_2 FC1-〖log〗_2 FC2|)/(〖|log〗_2 FC1+〖log〗_2 FC2|)

FC1 and FC2 represent the differential methylation fold changes in replicate 1 and replicate 2, respectively. Genomic regions with lower robust index are more credible in terms of differential methylation. We ranked the DMRs by robust index (Table S2), and re-analyzed both the top 500 and bottom 500 hypo DMRs. As shown in Figures S2b and S7 in our further revised manuscript, the analysis of both groups of DMRs supports our previous conclusions in the text, including the gradual decrease in DNA methylation during ripening, enrichment of DMRs in promoter regions, and decreased siRNA accumulation in the hypo DMRs during ripening.

We have lots of experience calling DMRs separately in each biological replicate and then determining the overlap between the DMRs of two biological replicates. However, we found that the overlapped DMRs between two biological replicates were not a very good representation of genome-wide methylation changes, since DMRs exclusive in replicate 1 also have significantly differential methylation levels in replicate 2, although they are not called DMRs in replicate 2 (probably due to cutoff and depth issues), and vice versa (e.g., Lang et al., PNAS 2017). Based on our experience, calling overlapped DMRs is not ideal in dealing with biological replicates. In several studies in the last several years (e.g., Moreno-Romero J. et al, the EMBO Journal 2016, 35(12):1298-1311; Tuncay Baubec et al, Cell 2013, 153(2):480-492; Watanabe T. et al, , the EMBO Journal 2018, 37(18); Saito Y et al, Nucleic Acids Research 2014, 42(6)), biological replicates were merged together for DMR calling, resulting in very reliable and accurate identification of DMRs. Following these previous studies and also based on our own experience, we merged the two biological replicates before DMR calling.
